# Identifying promoter features of co-regulated genes with similar network motifs

**DOI:** 10.1186/1471-2105-10-S4-S1

**Published:** 2009-04-29

**Authors:** Oscar Harari, Coral del Val, Rocío Romero-Zaliz, Dongwoo Shin, Henry Huang, Eduardo A Groisman, Igor Zwir

**Affiliations:** 1 Department of Computer Science and Artificial Intelligence, University of Granada, c/. Daniel Saucedo Aranda, s/n 18071, Granada, Spain; 2 Department of Molecular Cell Biology, Samsung Biomedical Research Institute, Sungkyunkwan University School of Medicine, Suwon 440-746, South Korea; 3 Department of Molecular Microbiology, Washington University School of Medicine, Campus Box 8230, 660 S. Euclid Ave., St. Louis, Missouri, 63110, USA; 4 Department of Molecular Microbiology, Washington University School of Medicine, Howard Hughes Medical Institute, Campus Box 8230, 660 South Euclid Avenue, St. Louis, Missouri, 63110-1093, USA

## Abstract

**Background:**

A large amount of computational and experimental work has been devoted to uncovering network motifs in gene regulatory networks. The leading hypothesis is that evolutionary processes independently selected recurrent architectural relationships among regulators and target genes (motifs) to produce characteristic expression patterns of its members. However, even with the same architecture, the genes may still be differentially expressed. Therefore, to define fully the expression of a group of genes, the strength of the connections in a network motif must be specified, and the *cis*-promoter features that participate in the regulation must be determined.

**Results:**

We have developed a model-based approach to analyze proteobacterial genomes for promoter features that is specifically designed to account for the variability in sequence, location and topology intrinsic to differential gene expression. We provide methods for annotating regulatory regions by detecting their subjacent *cis*-features. This includes identifying binding sites for a transcriptional regulator, distinguishing between activation and repression sites, direct and reverse orientation, and among sequences that weakly reflect a particular pattern; binding sites for the RNA polymerase, characterizing different classes, and locations relative to the transcription factor binding sites; the presence of riboswitches in the 5'UTR, and for other transcription factors. We applied our approach to characterize network motifs controlled by the PhoP/PhoQ regulatory system of *Escherichia coli *and *Salmonella enterica *serovar Typhimurium. We identified key features that enable the PhoP protein to control its target genes, and distinct features may produce different expression patterns even within the same network motif.

**Conclusion:**

Global transcriptional regulators control multiple promoters by a variety of network motifs. This is clearly the case for the regulatory protein PhoP. In this work, we studied this regulatory protein and demonstrated that understanding gene expression does not only require identifying a set of connexions or network motif, but also the *cis*-acting elements participating in each of these connexions.

## Background

Transcription regulatory networks can be represented as directed graphs in which a node stands for a gene (or an operon in the case of bacteria) and an edge symbolizes a direct transcriptional interaction. Recurrent patterns of interactions, termed network motifs, occur far more often than in randomized networks, forming elementary building blocks that carry out key functions. This is a convenient representation of the architecture of a set of regulatory Boolean (i.e. ON-OFF) networks, in which each gene is either fully expressed or not expressed at all, or that it has a binding site for a transcriptional regulator or lacks such a site. However, this approach has serious limitations because most genes are not expressed in a simple Boolean fashion. Indeed, genes that are co-regulated by the same transcription factor are often differently expressed with characteristic expression levels and kinetics. Therefore, a deeper understanding of regulatory networks demands the identification of the key features used by a transcriptional regulator to differentially control genes that display distinct behaviours despite belonging to networks with identical motifs.

The identification of the promoter features that determine the distinct expression behavior of co-regulated genes is a challenging task because: first, these features are often short combinations of a constrained four-symbol DNA alphabet. Therefore, it is not clear how to distinguish a sequence pattern that could affect gene expression from a just slightly different random sequence [1,2]. Second, the sequences recognized by a transcription factor may differ from promoter to promoter within and between genomes and may be located at various distances from other *cis*-acting features in different promoters [3,4]. Third, similar expression patterns can be generated from different or a mixture of multiple underlying features, thus, making it more difficult to discern the causes of analogous regulatory effects.

In this study, we present a method specifically aimed at handling the variability in sequence, location and topology that characterize gene transcription. We decompose a feature into a family of models or building blocks that uncover important differences among observations that are often concealed when using global patterns that tend to average sequences between promoters and even across species. This approach maximizes the sensitivity of detecting those instances that weakly resemble a consensus (e.g., binding site sequences) without decreasing the specificity. In addition, features are considered using fuzzy assignments, which allow us to encode how well a particular sequence matches each of the multiple models for a given promoter feature. Individual features can be linked into more informative composite models that can be used to explain the kinetic expression behavior of genes.

**Figure 1:**
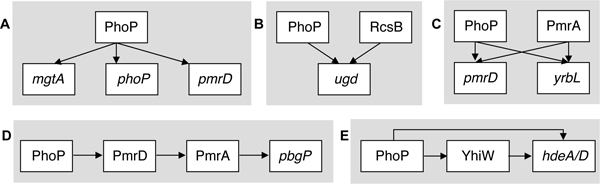
**The PhoP/PhoQ system employs a variety of network motifs to regulate gene transcription**. (A) In the single-input module, PhoP as a single transcription factor regulates a set of genes (i.e. *mgtA*, *phoP *and *pmrD*). (B) In the multi-input module, two or more transcription factors (e.g., PhoP and RcsB) regulate a target gene (i.e. *ugd*). (C) In the bi-fan module, a set of genes (i.e. *pmrD *and *yrbL*) are each regulated by a combination of transcription factors (i.e. PhoP and PmrA). (D) In the chained motif, genes are regulated in an ordered cascade. (E) In the feedforward loop, a transcription factor (i.e. PhoP) regulates the expression of a second transcription factor (i.e. YhiW), and both jointly regulate one or more genes (i.e. *hdeA/D*).

We applied our method to analyze promoters controlled by the PhoP/PhoQ regulatory system of *Escherichia coli *and *Salmonella enterica *serovar Typhimurium. This system responds to the same inducing signal (i.e. low Mg^2+^) in both species [4-7]. Moreover, the *E. coli phoP *gene could complement a *Salmonella phoP *mutant [8]. The DNA-binding PhoP protein appears to recognize a tandem repeat sequence separated by 5 bp [4-6], consistent with being a dimer [9]. The PhoP/PhoQ system is an excellent test case because it controls the expression of a large number of genes, amounting to ca. 3% of the genes in the case of *Salmonella *[10]. Furthermore, the PhoP/PhoQ regulon has been shown to employ a variety of network motifs including the single-input module (Fig. 1A), the multi-input module (Fig. 1B), the bi-fan (Fig. 1C), the chained (Fig. 1D), and the feedforward loop (Fig. 1E) [10-12]. Our analysis uncovered the salient features that distinguish genes co-regulated by PhoP belonging to similar networks. Gene transcription measurements provided experimental support for the investigated predictions.

## Results and discussion

### Approach

**Figure 2:**
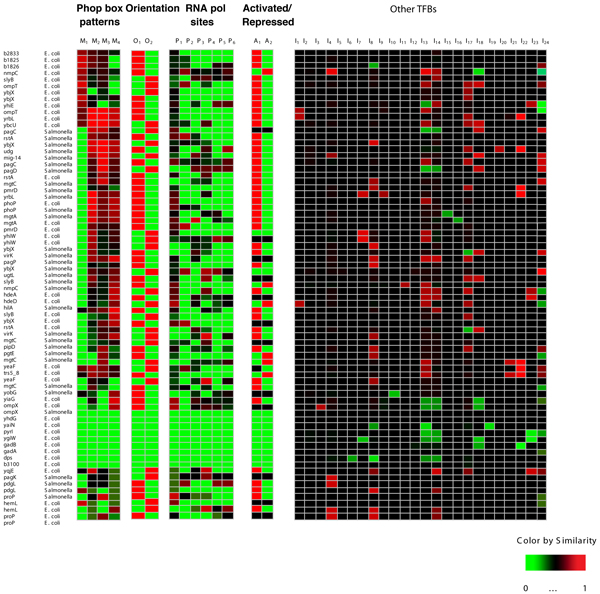
**PhoP-regulated promoters are described on the basis of five types of features**. We conform a database including whether the position of the PhoP box suggests that a promoter is activated or repressed (activated/repressed); the orientation of the PhoP box (orientation); distinct PhoP box patterns (motif patterns); the distance of the PhoP box relative to the RNA polymerase site and the class of sigma 70 promoter (RNA polymerase sites); and the presence of potential binding sites for 24 transcription factors in the PhoP-regulated promoters (Other TFBs). The identification of a feature in a promoter is based on measuring the degree of match between a promoter instance and a model that represents that feature, which results in a vector of [0, 1] values where 1 (red) corresponds to maximum matching and 0 (green) corresponds to the absence of the feature. Individual genes are allowed to have more than one promoter because more than one candidate PhoP box can be identified in an intergenic region. In addition, promoters for the same gene in different genomes are considered separately in the *E. coli *and *Salmonella *genomes. Activated/repressed analysis discriminates among three groups (A_1_-A_2_) corresponding to activated, and repressed genes, respectively. The PhoP box could be present in the opposite (O_1_) or the same (O_2_) orientation as the regulated open reading frame. Pattern analysis of the PhoP box resulted in four preliminary groups (M_1_-M_4_). RNA polymerase sites analysis revealed six groups (P_1_-P_6_) corresponding to types and location of sigma 70 promoters: (1) *close class II*, (2) *close class I*, (3) *medium class II*, (4) *medium class I*, (5) *remote class II *and (6) *remote class I*. The presence of other transcription factor binding sites in PhoP-regulated promoters includes: (1) OxyR, (2) FruR, (3) DeoR, (4) MalT, (5) MelR, (6) CytR, (7) GlpR, (8) ArcA, (9) FNR, (10) RcsB, (11) Fur, (12) ArgR, (13) RhaS, (14) AraC, (15) CRP, (16) DnaA, (17) YhiW, (18) Lrp, (19) NarL, (20) FIS, (21) IHF, (22) OmpR, (23) PmrA and (24) SlyA.

We investigated five types of *cis*-acting promoter features by extracting the maximal amount of useful information from datasets and then creating models that describe promoter regulatory regions. This entailed applying three key strategies: first, we conducted an initial survey of the data provided from different available sources, capturing and distinguishing between broad and easily discernable patterns. We then used these patterns as models to re-visit the data with greater sensitivity and specificity. This allowed us not only to recognize those instances with a low resemblance to consensus models, but also to reflect and annotate the diversity of the observations (*i.e*., when distances between the transcription factor binding site and RNA polymerase are unusual). Second, we utilized fuzzy clustering methods [13,14] to encode promoter matching to multiple models for a given promoter feature, which avoided having to make premature categorical assignments, and producing an initial classification of the promoters into multiple subsets. Finally, we applied fuzzy logic [15] to relate some basic features into more informative composite models that may explain the distinct expression behavior of genes belonging to similar networks (Fig. 2). A distinguishing characteristic of our approach is that promoters for orthologous genes are considered individually. This is in contrast to some phylogenetic footprinting methods [16] that often ignore regulatory differences among closely-related organisms due to their strict reliance on the conservation of regulatory motifs across bacterial species.

### Activated/repressed promoters

**Figure 3:**
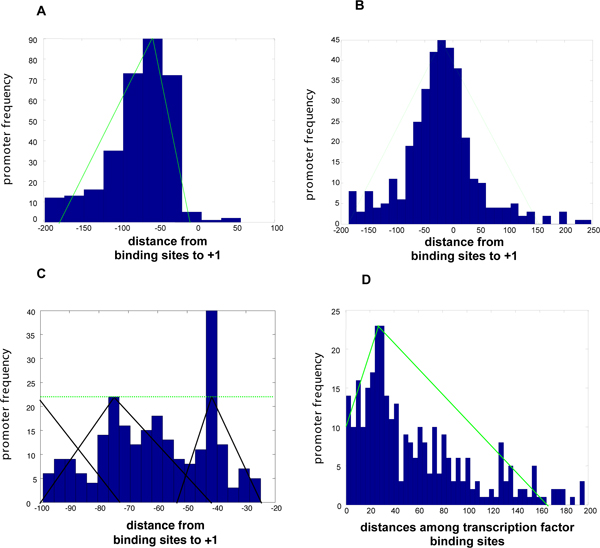
**Learning promoter features**. Promoter features were learned as models from examples in databases (e.g., RegulonDB) and then used to describe the intergenic regions of the *E. coli *and *S. enterica *genomes. (A, B) Promoters were classified into activated (A), repressed (B) or both, based on the location and the distance of a regulatory protein binding site to the RNA polymerase site. Different distributions are observed for activated, repressed and activated/repressed genes. The property that characterizes activated genes was learned from distances between the transcription start sites (+1) and the binding sites of different transcription factors. These distances were grouped in histograms and codified as elastic (fuzzy) functions, which can be interpreted as the membership degrees (in a unit interval) by which subsets of the dataset can embrace this property. (B) The histogram and membership function corresponding to repressed promoters. μ is maximal at much closer distances. Thus, the promoter distances can be probabilistically interpreted as the posterior probability *p*(*close*/*activated*) that given an *activated *gene, the regulator binding site is at a *close *distance from the transcription start site, following Bayes' rule. (C) The distances between transcription start sites (+1) and the binding sites of regulators were grouped into a histogram and codified as elastic (fuzzy)unit-interval functions. This process is analogous to fitting data from a parametric or non-parametric distribution and then assigning probabilities of membership to such distributions. We used these models to characterize the relationships between binding sites for the PhoP protein and the RNA polymerase binding site in the genome. Relationships were classified according to their similarity (fuzzy membership) with the prototypes to obtain a similarity vector of expression values. (D) The histogram illustrates the distances for binding sites of different regulators sharing the same promoter regions. The resulting membership functions, which were learned from such distributions, allows evaluating the putative relationship between a transcription factor motif and a PhoP box based both on motif quality and physical location.

Gene expression data normally allow clear separation of genes into those that are activated and those that are repressed by a regulatory protein. Because the expression signal is sometimes absent or too low to be informative, we considered the location of a transcription factor binding site relative to that of the RNA polymerase to separate promoters into activated and repressed subsets (Fig. 3A, B) [17].

We determined that the location of binding sites functioning in activation is different from that corresponding to sites functioning in repression (Fig. 3A, B), being centered ~40 and ~20 bp upstream of the transcription start site, respectively. This allowed us to distinguish among PhoP-regulated promoters that have apparently similar network motifs (Fig. 2). For example, we identified a PhoP binding site at a relative distance to the RNA polymerase consistent with repression in the promoter region of the *hilA *gene, which encodes a master regulator of *Salmonella *invasion and had been known to be under transcriptional repression by the PhoP/PhoQ system [18,19]. Several promoters, including those of the *Salmonella pipD *and *nmpC *genes, were classified as candidates for being both activated and repressed, because the distance between the predicted transcription start site and the PhoP box is consistent with either activation or repression. Gene expression experiments conducted in *E. coli *indicate that *nmpC *is a PhoP-repressed gene [4-6]. Other promoters were predicted to have more than one PhoP box (e.g., those of the PhoP-activated *mgtC *and *pagC *genes), where by their location one could correspond to an activation site and the other to a repression site [20].

### Transcription factor binding site orientation

**Figure 4:**
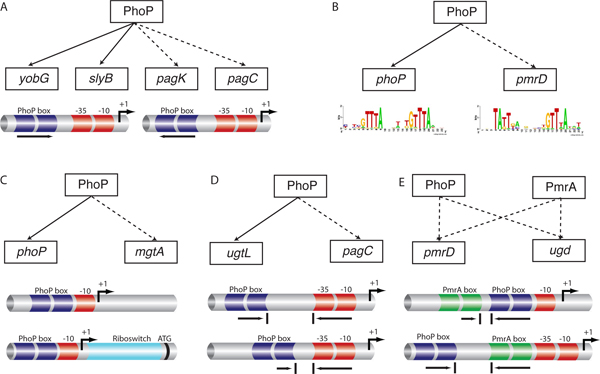
**The PhoP protein exhibits different *cis*-features for genes within the same network motif**. (A) PhoP-regulated promoters that differ in the orientation of the PhoP-binding site. PhoP regulates a set of promoters including those of the *Salmonella yobG*, *slyB*, *pagK *and *pagC *genes using a single-input network motif. We established that when *Salmonella *experiences low Mg^2+^, the PhoP protein binds to both the archetypal directly oriented *yobG *and *slyB *promoters as well as the oppositely oriented *pagK *and *pagC *promoters using chromatin immunoprecipitation (ChIP) *in vivo *[56]. (B) The PhoP protein uses the single-input network motif to control genes that differ in their binding site pattern. The PhoP protein recognizes a binding site motif consisting of a hexameric direct repeat separated by 5 bp, but distinguishes between different patterns with different specificities (i.e. *phoP *and *pmrD*). (C) PhoP regulates the *phoP *and *mgtA Salmonella *genes using the same network motif, however, *mgtA *harbors a riboswitch pattern in its 5'UTR region. (D) PhoP-regulated promoters differ in the RNA polymerase sites. The PhoP-activated *ugtL *and *pagC *promoters share the orientation of the PhoP-binding site as well as the class I sigma 70 promoter, but differ in the distance between the PhoP box and the RNA polymerase site. (E) Expression of PhoP-regulated promoters that use the bi-fan network motif. The *Salmonella pmrD*, and *ugd *promoters harbor experimentally verified PhoP- and PmrA-binding sites that can be described by the bi-fan network motif. The distance between the PhoP and PmrA boxes in the *Salmonella pmrD *and *ugd *promoters are also different (~38 bp and ~65 bp, respectively).

**Figure 5:**
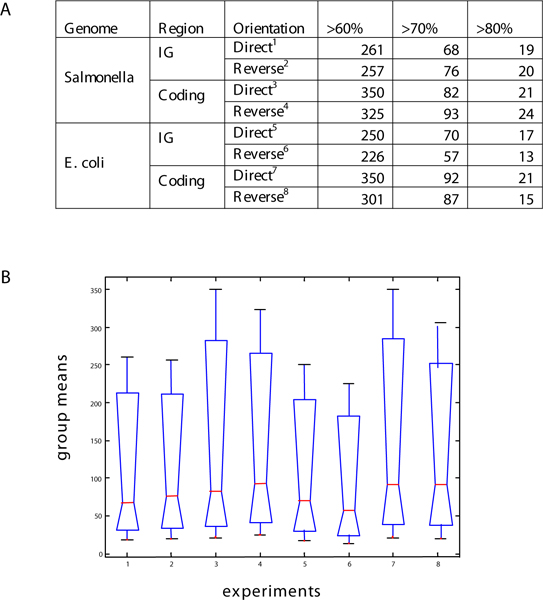
**Statistical significance of PhoP-binding site orientation**. (A) PhoP-binding sites were discovered in both possible orientations relative to the open reading frame, even though all published PhoP-binding sites are reportedly only in one (i.e. direct) orientation. To test the hypothesis that the genome harbors the PhoP-binding site in either orientation, we collected the number of PhoP-binding sites both in intergenic and coding regions of the *Salmonella *and *E. coli *genomes at different specificity levels. % indicates the relationship with the maximum score obtained by the Consensus/Patser program with a single consensus motif. Using a 95% confidence interval, we could not reject the null hypothesis using *one-way ANOVA*. (B) Multiple tests illustrates that we did not find significant differences in pairwise comparisons among six sets generated by splitting the data by regions, motif scores and genomes (we use Matlab *multicompare *routine with corrections for multiple tests). The horizontal axis corresponds to rows in (A), and the vertical axis illustrates the group means of the columns in (B).

Functional binding sites for a transcription factor may be present in either orientation relative to the RNA polymerase binding site [21]. This is due to the possibility of DNA looping and to the flexibility of the alpha subunit of the bacterial RNA polymerase in its interactions with transcriptional regulators [22,23]. Yet, promoters harboring binding boxes in different orientation can be controlled by PhoP using the same network motif. That is the case of the *yobG*, and *slyB *(direct), compared to *pagK *and *pagC *(opposite) *Salmonella *promoters (Fig. 4A). Analysis of PhoP-regulated promoters revealed that the PhoP box could be found with the same probability in either orientation in the intergenic regions of the *E. coli *and *Salmonella *genomes (Fig. 5). For example, the *E. coli ompT *and *yhiW *promoters and the *Salmonella mig-14, pipD*, *pagC *and *pagK *promoters harbor putative PhoP binding sites in the opposite relative orientation to that described for the prototypical PhoP-activated *mgtA *promoter [4] (Fig. 2). Yet other promoters (i.e. those of the *ybjX*, *slyB*, *yeaF *genes in *E. coli *and the *virK*, *ybjX*, and *mgtC *genes in *Salmonella*) contain sequences resembling the PhoP box in both orientations. The demonstration that PhoP does bind to the *mgtC, mig-14 *and *pagC *promoters [4], which harbor the PhoP binding site in the opposite orientation as in the *mgtA *promoter, validates our predictions and argues against alternative network designs where these promoters would be regulated by PhoP only indirectly [24].

### Transcription factor binding site patterns

Many genes are controlled by a single-input network motif where the affinity of a transcription factor for its promoter sequences is a major determinant of gene expression. Thus, co-regulated genes displaying distinct expression patterns are likely to differ in the binding site for such a transcription factor (Fig. 4B). Methods that look for matching to a sequence motif have been successfully used to identify promoters controlled by particular transcription factors [25-27]. However, the strict cutoffs used by such methods increase specificity but decrease sensitivity [26,28], which makes it difficult to detect binding sites with weak resemblance to a global sequence pattern [29].

We decomposed set of binding site sequences corresponding to a transcription factor into several patterns and then combined them to increased the sensitivity to weak sites without losing specificity (a detailed sensitivity performance analysis and evolutionary effects of these patters are described in O.H. *et al*, manuscript in preparation). In the case of PhoP, we used this approach to search both strands of the intergenic regions of the *E. coli *and *Salmonella *genomes (Fig. 2). This allowed the recovery of promoters, such as that corresponding to the *E. coli hdeA *gene or the *Salmonella pmrD*, that had not been detected by the single position weight matrix model [26,28] despite being footprinted by the PhoP protein [4-6,10-12]. The use of four patterns instead of a single consensus increased the sensitivity for PhoP binding sites from 46% to 74%; yet, the specificity remained essentially the same (i.e., 98% in a consensus model versus 97%). Importantly, this approach is not exclusive to binding sites recognized by the PhoP protein, but for other transcription factors reported in the RegulonDB database [30], where we could increase the sensitivity in an average of 35%, while retain almost the same sensitivity than a single position weight matrix (O.H. *et al*, manuscript in preparation).

### Riboswitch site patterns

Riboswitches are structured domains that usually reside in the non-coding regions of mRNAs (UTRs), where they bind specific metabolites and control gene expression. The most common effects occur at the level of premature termination of transcription (*cis*-acting) or translation initiation. Upstream regions of PhoP regulated genes were screened for riboswitches by analyzing the presence of segments with conserved secondary structure across genomes and thermodynamic stability; because Rfam  searches did not produce significant hits. Then, we evaluate if these candidate segments could be either small non-coding RNA or riboswitches, depending on their relative location to the beginning of the gene. Those candidates with conserved helixes, stable thermodynamically energy, and located close (<5 bp) to the translation start site of the closest gene, were further inspected as possible riboswitches. We found several genes with a long UTR region as possible candidates (see ). One of these genes is the *Salmonella mgtA *promoter, which has been experimentally validated (Fig. 4C) [31] showing that the DNA corresponding to a 264 nucleotide riboswitch confers Mg^2+ ^regulation when cloned in front of a reporter gene and behind a derivative of the *lac *promoter. Again, PhoP uses a similar network architecture to control promoters with differentially arranged regulatory regions (Fig. 4C).

### RNA polymerase binding site patterns and location

The distance of a transcription factor binding site to the RNA polymerase binding site(s) and the class of sigma 70 promoter are critical determinants of gene expression [22]. These classes correspond to the different types of contacts that can be established between a transcription factor and RNA polymerase. We identified six patterns among PhoP-regulated promoters of *E. coli *and *Salmonella *(Fig. 2) that combine promoter class and distance between the PhoP box and the RNA polymerase site (Fig. 3C). These patterns may correspond to a similar network motif, as it is the case of the *ugtL *and *pagC *promoters, which share the orientation of the PhoP box but differ in the distance of the PhoP box to the RNA polymerase binding site [22] (Fig. 4D).

Some PhoP-regulated promoters (e.g. the *hemL *and *phoP *promoters of *E. coli*) contain several putative RNA polymerase binding sites located at different positions and belonging to different classes, suggesting that such promoters may be regulated by additional signals and/or transcription factors [6]. The RNA polymerase site feature was evaluated using 721 RNA polymerase sites from RegulonDB as positive examples and 7210 random sequences as negative examples. We obtained an 82% sensitivity and 95% specificity for detecting RNA polymerase sites. These values provide a false discovery rate <0.001 and a correlation coefficient of 82%. In addition, we selected 34 examples of RNA polymerase sites reported to be of class II, which all differ from the typical class I promoter by exhibiting a degenerate -35 sequence motif [6,22,32], and obtained 74% sensitivity and 95% specificity.

### Binding sites for other transcription factors

Certain promoters harbor binding sites for more than one transcription factor. This could be because transcription requires the concerted action of such proteins, or because the promoter is independently activated by individual transcription factors, each responding to a distinct signal.

We analyzed the intergenic regions of the *E. coli *and *Salmonella *genomes for the presence of binding sites for 54 transcription factors [30]. We then investigated the co-occurrence of 24 sites with the binding site of the PhoP protein in an effort to uncover different types of network motifs involving PhoP-regulated promoters. For example, the *Salmonella pmrD*, *ugd *and *yrbL *promoters and the *E. coli yrbL *promoter harbor PhoP- and PmrA-binding sites, consistent with the experimentally-verified regulation by both the PhoP and PmrA proteins that can be described by the bi-fan network motif [4,33] (Fig. 4E). In addition, the relative position of transcription factor binding sites (Fig. 3D) can play a critical role because the PmrA-box in the *Salmonella pmrD *and *yrbL *promoters is located closer to the PhoP-box (~38 bp and ~24 bp, respectively) than in the *udg *promoter (~65 bp). By analyzing both the binding site quality and the location of transcription factor binding sites, we increase the chances of identifying co-regulated promoters.

By considering the presence of binding sites for multiple transcription factors, it is possible to generate hypotheses about potential network motifs. For example, the promoters of the PhoP-activated *gadA*, *dps*, *hdeA*, *yhiE *and *yhiW *genes of *E. coli *also have binding sites for the regulatory proteins YhiX and YhiE [4], raising the possibility that some of these genes might be regulated by feedforward loops where both the PhoP protein and either the YhiW or the YhiE proteins would bind to the same promoter to activate transcription. This notion was experimentally verified [4], validating our prediction.

### Evaluating the effect of distinct *cis*-regulatory features within a network motif

**Figure 6:**
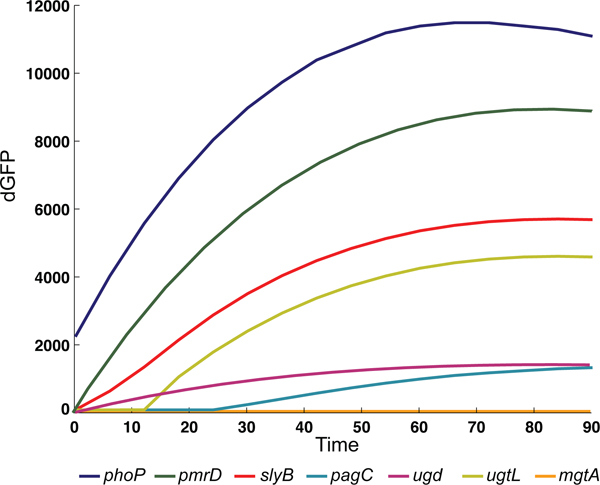
**Measurements of promoter activity and growth kinetics for GFP reporter strains with high-temporal resolution**. Transcriptional activity of wild-type *Salmonella *harboring plasmids with a transcriptional fusion between a promoterless *gfp *gene and the *Salmonella *promoters including *phoP *(blue), *pmrd*(green), *slyB *(red), *pagC *(cyan), *ugd*(magenta), *ugtL *(yellow) and *mgtA *(orange). Each experiment was conducted independently at least twice, and shown after preprocessing. The activity of each promoter is proportional to the number of GFP molecules produced per unit time per cell [*dG*_*i*_*(t)/dt]/OD*_*i*_*(t)*], where *G*_*i*_*(t) *is GFP fluorescence from wild-type *Salmonella *strain 14028s culture and conditions described in Methods, and *OD*_*i*_*(t) *is the optical density. The activity signal was smoothed by a polynomial fit (sixth order).

Gene expression is often measured by binary assays that evaluate differentials between wild-type and mutant strains (e.g., typical microarrays). These experiments always help to differentiate activated from repressed genes, and sometimes very low from very highly expressed genes. However, these approaches often conceal quantitative differences between true expressed genes. We hypothesize that distinct promoter features may affect gene expression even in similarly arranged network motifs. To test this notion, we compared the gene expression patterns of wild-type *Salmonella *harboring plasmids with a transcriptional fusion between a promoterless *gfp *gene to different PhoP-activated promoters (Fig. 6).

We found that promoters that differ in the orientation of the PhoP binding site and are arranged in a similar network motif such as *slyB *and *pagC *produce a complete different patterns of expression (Fig. 4A, 6). Moreover, single-output network motif including the *phoP *and the *pmrD *genes (Fig. 4B), which exhibit different PhoP box patterns, reveal a substantial different levels of promoter activity as measured by GFP kinetics (Fig. 6). Within the same network motif, we also evaluated the *mgtA *promoter and found that without specific primers for the 5'-UTR region the gene is unable to transcribe (Fig. 4C, 6). This suggests that the riboswitch located in the promoter region of *mgtA *is a critical feature that distinguishes promoters within the similar network (Fig. 4C). The *ugtL *and *pagC *promoters share the orientation and the PhoP box but differ in the distance of the PhoP box to the RNA polymerase binding site (Fig. 4D). This may account for the different kinetic behavior of these promoters when tested in a wild-type strain harboring plasmids with promoter fusions to the promoterless *gfp *gene (Fig. 6).

We also realized that the expression patterns differ in other types of network motifs such as the bi-fan. The Salmonella *pmrD *and *ugd *promoters harbour experimentally validated PhoP- and PmrA-boxes [10,34] (Fig. 4E), and both promoters confer distinct levels of expression as well as kinetic patterns (Fig. 6). Although it is hard to discern the specific and individual influence of each type of *cis*-feature, the preliminary results obtained by *gfp *experiments suggest that those regulatory elements described above can effectively produce differential gene expression even within similar network motifs.

## Conclusion

**Figure 7:**
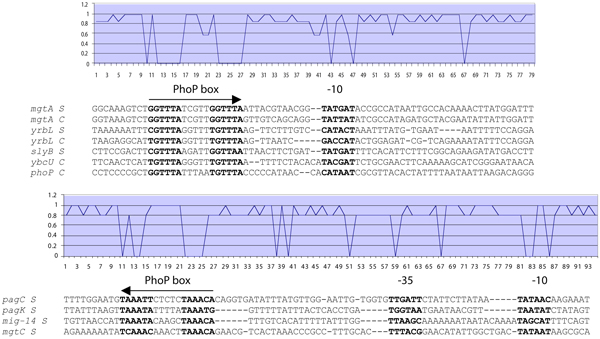
**Using promoter cis-features to annotate regulatory regions**. We recognized different PhoP binding box orientations and patterns, and RNA polymerase close class II and medium class I sites, and isolated the corresponding regions of promoters with similar features. Then, we described the similarity among DNA sequences in terms of the entropy of the frequency of the dominant base. This allowed us to visualize the variability of the promoter DNA sequences in terms of useful information (low values). These alignments with maximum information content could not be identified without using distinct *cis*-features harboring different patterns. This is clearly shown when the plain alignment of the intergenic region of all 11 promoters is performed (not shown).

We demonstrated that a transcription factor could mediate differential expression of genes described by the same network motif. This is because of the functional significance of variability in sequence, location and topology that exists among promoters that are co-regulated by a given transcription factor. We developed methods that encode and combine these promoter features, which allows matching of *cis*-observations to multiple models for a given promoter feature, into flexible databases constituting annotations of genome regulatory regions. These annotations cannot be uncovered by simpler sequence analysis approaches (Fig. 7). Indeed, the developed methods can be used to search and predict regulatory features even in incompletely characterized organism. Notably, these features do not constitute a computational artifact, but reflect different kinetic behaviours of co-regulated genes.

Global transcriptional regulators control multiple promoters by a variety of network motifs [27]. This is clearly the case for the regulatory protein PhoP (Fig. 1). In this work, we studied this regulatory protein and demonstrated that understanding gene expression does not only require identifying a set of connexions or network motif, but also the *cis*-acting elements participating in each of these connexions.

## Materials and methods

Our method consists of three phases: first, encoding the available information into preliminary model-based features, which includes identifying *cis*-features from DNA sequences and information from available databases; performing initial modeling of each individual feature, allowing the process of multiple occurrences of a feature and using relaxed thresholds and permitting missing values. A *model-based *feature is generated by the identification of a feature in a subset of observations (*F) *in the dataset, based on measuring the degree of match (*Q) *between an observation and a model, or a family of models (*M *= {*M*_*α*_}), at some degree (*α) *defined in a unit-interval scale (i.e., fuzzy values, *Q(F, M*_*α*_)) [35,36]. Second, grouping the results into subsets, thus, decomposing the preliminary models into a family of models or building blocks by using fuzzy clustering (see Additional file 1). Third, composing the building blocks by either combining the same or different types of features by using fuzzy logic expressions (see Additional file 1). And fourth, describing new promoters using the resulting models.

### Network motifs

In theory, the term "network motifs" is related to a statistical significant subgraph; however, in practice, they are treated as an over represented subgraph [37,38]. For example, a motif termed "single input motif " of three/four nodes in the *E. coli *(e.g., mfinder1.2 p-value < 34.7+-8.5) or *Saccharomyces cerevisiae *network [39] is not recognized as significant, while the only motif that exceeds the standard threshold is the "feed forward motif".

### Activated/repressed

We modeled PhoP-regulated promoters as activated or repressed based on examples reported in the RegulonDB database [30]. **(1) **We separately grouped activated and repressed promoters, and plotted histograms for each group corresponding to the distances between transcription factor binding sites and the transcription initiation (+1) site. **(2) **We distinguished two non-disjoint distributions in each group and built models for these distances by fitting histograms with fuzzy membership functions [15] (Fig. 3A, B) (see Additional file 1), which do not force promoters to be exclusively Activated or Repressed. **(3) **Finally, we connected **(2) **and sigma 70 promoters previously detected to select the most representative candidate for each promoter condition (e.g., best promoter that characterize the activated condition) by using fuzzy logic-based operations (see Additional file 1), which also have a probabilistic interpretation (e.g., *p*(*activated*/*sigma *70)), to characterize relationships between predicted PhoP and RNA polymerase binding sites detected in candidate promoters (see below). Simple features, such as activated and repressed can be combined in more complex composite models to represent divergently transcribed genes (e.g., two adjacent genes, one repressed, the other activated, both sharing the same putative PhoP box in different orientations) using fuzzy logic expressions (see Additional file 1).

### Binding site patterns and orientation

**(1) **We built an initial model for the PhoP binding site by learning a position weight matrix [28] (*E-value *< 10E-12) based on the upstream sequences of genes corresponding to the training set of the *E. coli *and *Salmonella *genomes (Table S1, Additional file 1). **(2) **We searched the intergenic regions of the genes in both orientations, using low thresholds corresponding to two standard deviations below the mean score obtained with the initial model [40]. Multiple PhoP binding site candidates were allowed in a given promoter operator region. **(3) **After transforming nucleotides into dummy variables [41], we grouped sequences matching the PhoP position weight matrix using the fuzzy C-means clustering method with the Xie-Beni validity index (see Additional file 1) to estimate the number of clusters [13,42]. **(4) **We built models for these clusters using position weight matrices (*E-value *< 10E-22) and searched the *E. coli *and *Salmonella *genomes to characterize each gene according to its similarity to each model as a fuzzy partition (Fig. 2).

### Riboswitch site patterns

**(1) **We employed upstream regions of PhoP regulated genes to create conserved sequence aligments by comparisons against representative proteobacterial genomes. We used WU BLAST 2.0 [43] with a word hit of eight, and using default parameters otherwise. **(2) **We selected alignments with an *E*-value ≤ 0.00001 and a length ≥ 50 nt; and divided alignments longer than 300 bp into windows of 300 bp with 50 bp of overlap. **(3) **These windows fed the programs eQRNA and RNAz following the protocol described in [44] using a window size of 200 nucleotides and a window slide increment of 50 nucleotides. QRNA analysis was performed with eQRNA version 2.0.3c. (). **(3.1) **We classified the alignment as RNA, coding, or other, according to the Bayesian posterior probability of each model. RNAz was used with its version 0.1.1 . We only considered overlapping eQRNA and RNAz predictions for the upstream regions of PhoP regulated genes as candidates for small non-coding RNA or riboswitches. (4) We encoded the conservation identity of the segments and their distance to the translation start site of the closest gene as fuzzy sets; and aggregated them using fuzzy expressions (see Additional file 1). **(5) **All fuzzy expressions of a single gene were combined using the Maximum T-conorm (see Additional file 1).

### RNA polymerase sites

**(1) **We gathered sigma 70 class I and class II promoters [32,45] from the RegulonDB database and [46]. Then, we built models of the RNA polymerase site using a neuro-fuzzy method (see HPAM in [47]), and used the resulting models to perform genome-wide descriptions of the intergenic regions of the *E. coli *and *Salmonella *genomes with a false discovery rate <0.001 (see Promoter search in ). **(2) **We used an intelligent parser to differentiate class I and class II promoters that evaluate the quality of the -35 motif [22,32], based on fuzzy logic (see Additional file 1) and genetic algorithms techniques (see MOSS in gps-tools2.wustl.edu [48]). **(3) **To characterize the distance relationship between transcription factors binding sites and RNA polymerase binding sites, we built models of such distances from the examples reported in the RegulonDB database. **(3.1) **We modeled activated and repressed promoters (see below *Activated or repressed *feature). **(3.2) **We re-built histograms for each group of distances (i.e. activated and repressed), distinguishing three overlapping distributions for each of them.**(3.3) **We built models for distances by fitting their distributions into models based on fuzzy membership functions [15] (see Additional file 1), which were termed close, medium and remote distances for each set of activated and repressed genes (Fig. 3C). Finally, to characterize the distance relationship between the PhoP box and putative RNA polymerase binding site, we connected (2) and (3) by using fuzzy logic-based operations (see Additional file 1).

This process allowed us to retrieve the most representative RNA polymerase binding site candidates for each promoter region relative to the PhoP binding site (e.g., best class II RNA polymerase site, which is located close to the PhoP box in an activated promoter), which were arrayed and constituted the value of the RNA polymerase site feature in Fig. 2. The probabilistic interpretation of the former process is usually the posterior probability (e.g., *p*(*class II*/*close*) that, given a *close *promoter, it comes from class "class II" by following Bayes' rule [13,41,42]). This process is analogous to classification methods termed Naïve Bayes [49] if the T-norm and the T-conorm (see Additional file 1) are restricted to the Product and the Maximum.

### Binding sites for other transcription factors

We developed models for different transcription factor binding sites from the RegulonDB database as follows: **(1) **We built position weight matrices for each transcription factor using the Consensus/Patser program, choosing the best final matrix for motif lengths between 14–30 bps if the corresponding length had not been previously specified (see "Consensus matrices" in ). We accounted for the motif symmetry (e.g., asymmetric, direct, inverted [45]) if available (see "Search known transcription factor motifs" in ). **(2) **We searched the intergenic regions of the *E. coli *and *Salmonella *genomes with these models, using the correlation coefficient measure (see Additional file 1) and additional 772 promoters from the RegulonDB database [30] to establish a threshold (average *E-value *< 10E-10) for each matrix [50] (see "Thresholded consensus" in ). **(3) **We accounted for the distances between distinct transcription factors binding sites occurring in the same promoter region (e.g., the distance between the CRP and FIS sites in the *proP *promoter [51]) in promoters reported in RegulonDB database and built a histogram with the obtained results (Fig. 3D). **(4) **We fitted the histogram using a fuzzy membership function (see Additional file 1) and used this model as a fuzzy cluster to characterize the distances between a putative PhoP box and another putative transcription factor binding site detected in the same region. **(5) **Finally, we connected **(2) **and **(4) **by using fuzzy logic-based operations (see Additional file 1), which can also have a probabilistic interpretation (e.g., *p(CRP, FIS/appropriate distance*) upstream of the *proP *open reading frame of *E. coli*), to characterize PhoP regulated candidates promoters.

### Dataset

We initially used the intergenic regions of *E. coli *and *Salmonella *operons from -800 to +50 because > 5% are larger than 800 bp in bacterial genomes (as described in the RegulonDB database or generously provided by H. Salgado) [49]; however, predictions have been performed in whole coding and non coding regions (see ). The promoter and transcription factor information was taken from RegulonDB database. We compiled from the literature and our own lab information (Table S1, Additional file 1) genes whose expression (using microarrays) differed statistically between wild-type and *phoP E. coli *strains experiencing inducing conditions for the PhoP/PhoQ regulatory system [4], as well as a list of genes known/assumed to be PhoP regulated [52]. However, this information did not explicitly indicate whether these genes were regulated directly or indirectly by the PhoP protein. The learned features were used to make genome-wide predictions in the *E. coli *and *Salmonella *genomes.

### Programming resources

The scripts and programs used in this work, some of which are accessible from  web site, were based on Perl, Matlab r2006a and C++ interpreters/languages, and the visualization routines were performed on Spotfire DecisionSite software 8.2. Data and predictions for *E. coli *and *Salmonella *genomes are available at supplemental table S1 in Additional file 1 and at .

### Bacterial strains, plasmids and growth conditions

Bacterial strains and plasmids used in this study are listed in Table S2, Additional file 1. *Salmonella enterica *serovar Typhimurium strains used in this study are derived from strain 14028s. Bacteria were grown at 37°C in Luria-Bertani broth (LB) [53] or N-minimal medium pH 7.7 [54] supplemented with 0.1% Casamino Acids, 38 mM glycerol, MgCl_2_. Kanamycin was used at 25 μg/ml.

### Constructions of GFP reporter plasmids

Promoter regions (i.e. the intergenic region between two ORFs) were amplified using PCR. A list of the promoter-specific primers used in the PCR reactions is shown in Table S3, Additional file 1. The PCR fragment was digested with *Bam*HI and *Xho*I, purified, then introduced to the cloning site of pMS201 (GFP reporter vector plasmid, a gift from Alon, U. [55]). Sequences of promoter region were verified by nucleotide sequencing.

### Measurements of promoter activity and growth kinetics for GFP reporter strains

Promoter activity and growth kinetics of wild-type *Salmonella *strain harboring GFP reporter plasmid was measured in parallel using automated microplate reader (VICTOR^3^, Perkin Elmer) [55]. Overnight cultures of strains in N-minimal medium with 10 mM MgCl_2 _and 25 μg/ml of kanamycin were washed with the same medium without MgCl_2 _then diluted (1:100) to 96-well plate (Packard) containing 150 μl of N-minimal media supplemented 50 μM MgCl_2_. After overlaying the wells with 50 μl of mineral oil (Sigma) to prevent evaporation of media, the plate was inserted in the VICTOR^3 ^machine pre-warmed to 37°C. The fluorescence and optical density (600 nm) of cells were recorded with shaking of the plate (1 min with 0.1 mm diameter), and this protocol was repeated every 6 min for 99 times. The background fluorescence was measured using a strain carrying empty vector and subtracted from the test values. Each experiment was conducted independently twice, and a representative is shown in the figures.

### Data preprocessing

The raw GFP and OD signals were used to calculate the promoter activity as [*dG*_*i*_*(t)/dt*]/*OD*_*i*_*(t)*. The activity signal was then smoothed by a shape-preserving interpolant (Piecewise Cubic Hermite Interpolating Polynomial, Matlab r2006a) fitting algorithm that finds values of an underlying interpolating function at intermediate points that are not described in the experimental assays. Then, we applied a polynomial fit (sixth order, Matlab r2006a) on each expression signal. This smoothing procedure captures the dynamics well, while removing the noise inherent in the differentiation of noisy signals.

## Competing interests

The authors declare that they have no competing interests.

## Authors' contributions

OH and IZ designed and implemented the methods and wrote the manuscript; CV designed and implemented the riboswitch identification methods; RRZ coded the perl scripts and the web page; DS performed the experimental validation using GFP technology; HH provided advice on the project and revised the manuscript; EAG supported the project and drafted the manuscript.

## Supplementary Material

Additional File 1 **Supplemental tables**. Table S1 provide the features describing PhoP regulated promoters and raw data used to build them. Table S2 details the bacterial strains and plasmids used in this study, and Table S3 the primers used to construct the promoters in GFP reporter plasmids. Click here for file

## References

[B1] Beer MA, Tavazoie S (2004). Predicting gene expression from sequence. Cell.

[B2] Pritsker M, Liu YC, Beer MA, Tavazoie S (2004). Whole-genome discovery of transcription factor binding sites by network-level conservation. Genome Res.

[B3] Winfield MD, Groisman EA (2004). Phenotypic differences between Salmonella and Escherichia coli resulting from the disparate regulation of homologous genes. Proc Natl Acad Sci USA.

[B4] Zwir I, Shin D, Kato A, Nishino K, Latifi T, Solomon F, Hare JM, Huang H, Groisman EA (2005). Dissecting the PhoP regulatory network of Escherichia coli and Salmonella enterica. Proc Natl Acad Sci USA.

[B5] Eguchi Y, Okada T, Minagawa S, Oshima T, Mori H, Yamamoto K, Ishihama A, Utsumi R (2004). Signal Transduction Cascade between EvgA/EvgS and PhoP/PhoQ Two-Component Systems of Escherichia coli. J Bacteriol.

[B6] Minagawa S, Ogasawara H, Kato A, Yamamoto K, Eguchi Y, Oshima T, Mori H, Ishihama A, Utsumi R (2003). Identification and molecular characterization of the Mg2+ stimulon of Escherichia coli. J Bacteriol.

[B7] Soncini FC, Garcia Vescovi E, Solomon F, Groisman EA (1996). Molecular basis of the magnesium deprivation response in Salmonella typhimurium: identification of PhoP-regulated genes. J Bacteriol.

[B8] Groisman EA, Heffron F, Solomon F (1992). Molecular genetic analysis of the Escherichia coli phoP locus. J Bacteriol.

[B9] Perron-Savard P, De Crescenzo G, Le Moual H (2005). Dimerization and DNA binding of the Salmonella enterica PhoP response regulator are phosphorylation independent. Microbiology.

[B10] Kato A, Latifi T, Groisman EA (2003). Closing the loop: the PmrA/PmrB two-component system negatively controls expression of its posttranscriptional activator PmrD. Proc Natl Acad Sci USA.

[B11] Mouslim C, Latifi T, Groisman EA (2003). Signal-dependent requirement for the co-activator protein RcsA in transcription of the RcsB-regulated ugd gene. J Biol Chem.

[B12] Shi Y, Latifi T, Cromie MJ, Groisman EA (2004). Transcriptional control of the antimicrobial peptide resistance ugtL gene by the Salmonella PhoP and SlyA regulatory proteins. J Biol Chem.

[B13] Bezdek JC, Pedrycz W, Bonissone PP, Ruspini EH (1998). Pattern Analysis. Handbook of Fuzzy Computation.

[B14] Gasch AP, Eisen MB (2002). Exploring the conditional coregulation of yeast gene expression through fuzzy k-means clustering. Genome Biol.

[B15] Klir GJ, Folger TA (1988). Fuzzy sets, uncertainty, and information.

[B16] McCue L, Thompson W, Carmack C, Ryan MP, Liu JS, Derbyshire V, Lawrence CE (2001). Phylogenetic footprinting of transcription factor binding sites in proteobacterial genomes. Nucleic Acids Res.

[B17] Collado-Vides J, Magasanik B, Gralla JD (1991). Control site location and transcriptional regulation in Escherichia coli. Microbiol Rev.

[B18] Groisman EA (2001). The pleiotropic two-component regulatory system PhoP-PhoQ. J Bacteriol.

[B19] Schechter LM, Damrauer SM, Lee CA (1999). Two AraC/XylS family members can independently counteract the effect of repressing sequences upstream of the hilA promoter. Mol Microbiol.

[B20] Tu X, Latifi T, Bougdour A, Gottesman S, Groisman EA (2006). The PhoP/PhoQ two-component system stabilizes the alternative sigma factor RpoS in Salmonella enterica. Proc Natl Acad Sci U S A.

[B21] Lobell RB, Schleif RF (1991). AraC-DNA looping: orientation and distance-dependent loop breaking by the cyclic AMP receptor protein. J Mol Biol.

[B22] Barnard A, Wolfe A, Busby S (2004). Regulation at complex bacterial promoters: how bacteria use different promoter organizations to produce different regulatory outcomes. Curr Opin Microbiol.

[B23] Teichmann SA, Babu MM (2002). Conservation of gene co-regulation in prokaryotes and eukaryotes. Trends Biotechnol.

[B24] Lejona S, Aguirre A, Cabeza ML, Garcia Vescovi E, Soncini FC (2003). Molecular characterization of the Mg2+-responsive PhoP-PhoQ regulon in Salmonella enterica. J Bacteriol.

[B25] Bailey TL, Elkan C (1995). The value of prior knowledge in discovering motifs with MEME. Proc Int Conf Intell Syst Mol Biol.

[B26] Stormo GD (2000). DNA binding sites: representation and discovery. Bioinformatics.

[B27] Martinez-Antonio A, Collado-Vides J (2003). Identifying global regulators in transcriptional regulatory networks in bacteria. Curr Opin Microbiol.

[B28] Hertz GZ, Stormo GD (1999). Identifying DNA and protein patterns with statistically significant alignments of multiple sequences. Bioinformatics.

[B29] Tompa M, Li N, Bailey TL, Church GM, De Moor B, Eskin E, Favorov AV, Frith MC, Fu Y, Kent WJ (2005). Assessing computational tools for the discovery of transcription factor binding sites. Nat Biotechnol.

[B30] Salgado H, Gama-Castro S, Martinez-Antonio A, Diaz-Peredo E, Sanchez-Solano F, Peralta-Gil M, Garcia-Alonso D, Jimenez-Jacinto V, Santos-Zavaleta A, Bonavides-Martinez C (2004). RegulonDB (version 4.0): transcriptional regulation, operon organization and growth conditions in Escherichia coli K-12. Nucleic Acids Res.

[B31] Cromie MJ, Shi Y, Latifi T, Groisman EA (2006). An RNA sensor for intracellular Mg(2+). Cell.

[B32] Ishihama A (1993). Protein-protein communication within the transcription apparatus. J Bacteriol.

[B33] Kato A, Groisman EA (2004). Connecting two-component regulatory systems by a protein that protects a response regulator from dephosphorylation by its cognate sensor. Genes Dev.

[B34] Mouslim C, Groisman EA (2003). Control of the Salmonella ugd gene by three two-component regulatory systems. Mol Microbiol.

[B35] Ruspini EH, Zwir I, Pal SK, Pal A (2002). Automated generation of qualitative representations of complex objects by hybrid soft-computing methods. Pattern recognition: from classical to modern approaches.

[B36] Zwir I, Zaliz RR, Ruspini EH (2002). Automated biological sequence description by genetic multiobjective generalized clustering. Ann N Y Acad Sci.

[B37] Shen-Orr SS, Milo R, Mangan S, Alon U (2002). Network motifs in the transcriptional regulation network of Escherichia coli. Nat Genet.

[B38] Milo R, Shen-Orr S, Itzkovitz S, Kashtan N, Chklovskii D, Alon U (2002). Network motifs: simple building blocks of complex networks. Science.

[B39] Lee TI, Rinaldi NJ, Robert F, Odom DT, Bar-Joseph Z, Gerber GK, Hannett NM, Harbison CT, Thompson CM, Simon I (2002). Transcriptional regulatory networks in Saccharomyces cerevisiae. Science.

[B40] Robison K, McGuire AM, Church GM (1998). A comprehensive library of DNA-binding site matrices for 55 proteins applied to the complete Escherichia coli K-12 genome. J Mol Biol.

[B41] Everitt B, Der G (1996). A handbook of statistical analysis using SAS.

[B42] Bezdek JC, Pal SK, IEEE Neural Networks Council (1992). Fuzzy models for pattern recognition : methods that search for structures in data.

[B43] Lopez R, Silventoinen V, Robinson S, Kibria A, Gish W (2003). WU-Blast2 server at the European Bioinformatics Institute. Nucleic Acids Res.

[B44] del Val C, Rivas E, Torres-Quesada O, Toro N, Jimenez-Zurdo JI (2007). Identification of differentially expressed small non-coding RNAs in the legume endosymbiont Sinorhizobium meliloti by comparative genomics. Mol Microbiol.

[B45] Salgado H, Santos-Zavaleta A, Gama-Castro S, Millan-Zarate D, Diaz-Peredo E, Sanchez-Solano F, Perez-Rueda E, Bonavides-Martinez C, Collado-Vides J (2001). RegulonDB (version 3.2): transcriptional regulation and operon organization in Escherichia coli K-12. Nucleic Acids Res.

[B46] Harley CB, Reynolds RP (1987). Analysis of E. coli promoter sequences. Nucleic Acids Res.

[B47] Cotik V, Zaliz RR, Zwir I (2005). A hybrid promoter analysis methodology for prokaryotic genomes. Fuzzy Sets and Systems.

[B48] Romero Zaliz R, Zwir I, Ruspini EH, Coello Coello CaL G (2004). Generalized analysis of promoters: a method for DNA sequence description. Applications of Multi-Objective Evolutionary Algorithms.

[B49] Mitchell TM (1997). Machine learning.

[B50] Benitez-Bellon E, Moreno-Hagelsieb G, Collado-Vides J (2002). Evaluation of thresholds for the detection of binding sites for regulatory proteins in Escherichia coli K12 DNA. Genome Biol.

[B51] McLeod SM, Aiyar SE, Gourse RL, Johnson RC (2002). The C-terminal domains of the RNA polymerase alpha subunits: contact site with Fis and localization during co-activation with CRP at the Escherichia coli proP P2 promoter. J Mol Biol.

[B52] Zwir I, Huang H, Groisman EA (2005). Analysis of Differentially-Regulated Genes within a Regulatory Network by GPS Genome Navigation. Bioinformatics.

[B53] Sambrook J, Fritsch EF, Maniatis T (1989). Molecular cloning: a laboratory manual.

[B54] Snavely MD, Gravina SA, Cheung TT, Miller CG, Maguire ME (1991). Magnesium transport in Salmonella typhimurium. Regulation of mgtA and mgtB expression. J Biol Chem.

[B55] Mangan S, Alon U (2003). Structure and function of the feed-forward loop network motif. Proc Natl Acad Sci USA.

[B56] Shin D, Groisman EA (2005). Signal-dependent Binding of the Response Regulators PhoP and PmrA to Their Target Promoters in Vivo. J Biol Chem.

