# Supplementary material for: Identifying promoter features of co-regulated genes with similar network motifs
Source: BMC Bioinformatics. 2009 Apr 29;10(Suppl 4):S1. doi: 10.1186/1471-2105-10-S4-S1 (PMC2681069; doi:10.1186/1471-2105-10-S4-S1)
Supplement: Additional File 1 — Supplemental tables. Table S1 provide the features describing PhoP regulated promoters and raw data used to build them. Table S2 details the bacterial strains and plasmids used in this study, and Table S3 the primers used to construct the promoters in GFP reporter plasmids. [file 1471-2105-10-S4-S1-S1.pdf]

Identifying promoter features of co-regulated genes with similar network motifs

Supplemental Text

Fuzzy sets and logic expressions

Fuzzy logic expression incorporate predicates having fuzzy variables, which are manipulated using various theorems/axioms and methods [1]. These expressions have been widely applied to model relational clusters or features [1, 2]. Given a dataset $X = \{x_1,...,x_n\}$, the feature that characterizes it can be best described as a set $F_1(X) = \{d_{11}/x_1,...,d_{1n}/x_n\}$, where $\{d_{11},...,d_{1n}\} \in \{0,1\}$ in classical set theory and $[0,1]$ in fuzzy set theory. These fuzzy values represent the degree of matching between an observation of the dataset and a fuzzy set defined using triangular functions [1]:

$$\mu(x) = \begin{cases} 0 & \text{if } x < a_0 \text{ or } x > a_2 \\ (x - a_0)/(a_1 - a_0) & \text{if } x < a_1 \\ (a_2 - x)/(a_2 - a_1) & \text{if } x > a_1 \\ 1 & \text{otherwise} \end{cases} \quad (1)$$

where $a_0,...,a_2$ are learned from the projection of the histograms onto the variable domains by simple regression and minimum squared methods [3, 4]. This process is analogous to fitting histograms to a distribution, and assigning probability values based on a density function. Our approach, however, adopts a distribution-independent and non-parametric fitting process by projecting data [3, 4] into triangular functions. The degree of matching is defined in the unit interval and can be obtained from evaluating the membership function of the corresponding fuzzy set. Then, given $F_2(X) = \{d_{21}/x_1,...,d_{2n}/x_n\}$ and the Minimum as a T-norm (intersection) operator:

$$F_1(X)\, AND\, F_2(X) = F_1 \cap F_2 = MIN(F_1, F_2) = \{MIN(d_{11}, d_{21})/x_1, ..., MIN(d_{1n}, d_{2n})/x_n\}$$

Combination of several expressions is performed by using the Maximum as a T-conorm (union) operator.

Fuzzy C-Means clustering method

This method [5, 6] is an extension of the *K*-means clustering method, where the elements can belong to more than one cluster with a different degree of membership. Thus, membership of a promoter *k* with a feature value x_k in a particular cluster V_i is calculated as:

$$\mu_{i,k} = \left[1 + \left(\left\|x_k - V_i\right\|_A^2 / w_i\right)^{1/m-1}\right]^{-1} \quad \forall i,k;\ 1 < m \le \infty \quad (2)$$

where *c*-partitions of the data *X* were usually arrayed as a ($c \times n$) matrix U containing the vector representation of matching between *n* promoters and *c*-partitions of one type of feature, $\mu_{i,k}$ is taken as the degree of membership of the value x_k in the *i*th partitioning fuzzy subset of *X*, $\overline{V_i}$ is the cluster prototype or centroid of partition V_i, *m* is the degree of fuzzification, A determines the type of norm commonly used in pattern recognition (e.g., *A*=1 is the city block norm; *A*=2 is the Euclidean norm, etc [2]), and a weight for penalty terms w_i, which is initialized as 1 in the absence of external information. If the approach is probabilistic $\mu_{i,k}$ is usually the posterior probability $p(i/x_k)$ that, given x_k, it comes from class *i* by following Bayes' rule [2, 6, 7]. If the approach is fuzzy, x_k can come from more than one class, and if it is possibilistic [2], a more realistic situation is

represented where we do not force each element to belong to a class. The cluster prototype or centroid of partition V_i is calculated as:

$$V_i = \frac{\sum_{k=1}^{n} (\mu_{i,k})^m x_k}{\sum_{k=1}^{n} (\mu_{i,k})^m} \quad \forall i \quad (3)$$

based on the use of the Euclidean distance as a similarity function:

$$\|x, V\|_2 = \sqrt{(x-V)^T (x-V)} \quad (4)$$

Summarizing, (0) Initialize $V_0 = \{v_1, ..., v_c\}$ (1) While (t < T and $\|V_t - V_{t-1}\| > \varepsilon$) (2) Calculate U_t with V_{t-1}, (3) update V_{t-1} to V_t, with U_t (4) Iterate, where T is the maximum number of iterations. The minimization of this index [2] through different number of clusters (i.e., $c = 2$ to $c = \sqrt{n}$) detects compact representations of Fuzzy C-Means partitions:

$$XB(U,L) = \frac{\sum_{k=1}^{n} \sum_{i=1}^{c} u_{i,k}^2 \|x_k - \overline{V_i}\|^2}{n\left(\min_{i \neq j} \left\{ \|\overline{V_i} - \overline{V_j}\|^2 \right\} \right)} \quad (4)$$

Performance Measurement

We use a correlation coefficient implementation to establish best local thresholds for transcription factor binding site motifs. That is, from a range of possible thresholds applied over a particular motif, we choose the one that maximizes this coefficient defined as: $CC = (TP \times TN) - (FP \times FN) / \sqrt{(TP + FP) \times (TN + FN) \times (TP + FN) \times (TN + FP)}$, where specificity = TN/(TN + FP) and sensitivity = TP/(TP + FN); P= positive, N=negative, T = true and F=false [8]. We constrained the sensitivity of the selected threshold to be above the 60%. The false positive rate for binding site analysis was

calculated by detecting binding sites from other transcription factors different from the one being evaluated (RegulonDB database).

References

1. Klir GJ, Folger TA: **Fuzzy sets, uncertainty, and information.** London: Prentice Hall International; 1988.
2. Bezdek JC: **Pattern Analysis**. In: *Handbook of Fuzzy Computation.* Edited by Pedrycz W, Bonissone PP, Ruspini EH. Bristol: Institute of Physics; 1998: F6.1.1-F6.6.20.
3. Everitt B, Der G: **A handbook of statistical analysis using SAS**. London: Chapman & Hall; 1996.
4. Sugeno M, Yasukama T: **A Fuzzy-logic-based Approach to Qualitative Modeling**. *IEEE Transactions on Fuzzy Systems* 1993, **1**(1):7-31.
5. Gasch AP, Eisen MB: **Exploring the conditional coregulation of yeast gene expression through fuzzy k-means clustering**. *Genome Biol* 2002, **3**(11):RESEARCH0059.
6. Bezdek JC, Pal SK, IEEE Neural Networks Council: **Fuzzy models for pattern recognition : methods that search for structures in data**. New York: IEEE Press; 1992.
7. Mitchell TM: **Machine learning.** New York: McGraw-Hill; 1997.
8. Benitez-Bellon E, Moreno-Hagelsieb G, Collado-Vides J: **Evaluation of thresholds for the detection of binding sites for regulatory proteins in Escherichia coli K12 DNA**. *Genome Biol* 2002, **3**(3):RESEARCH0013.

Supplemental Tables

Table S1: Features describing PhoP regulated promoters and raw data used to build them.

					PhoP-Box pattern					Orientation	
										Direct	***Opposite***
Gene	***Code***	***Genome***	***Philogeny***	***Identity***	***M1***	***M2***	***M3***	***M4***	***Sequence***	***O1***	***O2***
b2833	73	E. coli	ygdR	90.28	0.788	0.639	0.578	0.422	AGATATATAACGTCGGTTTATAA	1	0
b1825	6	E. coli	STM1839	63.16	0.914	0.699	0.608	0.000	ACATAGTTAGGCGCTGTTTAACT	1	0
b1826	28	E. coli	yobG	65.96	0.914	0.699	0.608	0.000	ACATAGTTAGGCGCTGTTTAACT	1	0
nmpC	221	E. coli	nmpC	81.71	0.809	0.521	0.586	0.422	TGTTATTTTTAGCCGGTTTAAAT	1	0
slyB	351	E. coli	slyB	61.94	0.791	0.428	0.586	0.195	TGGTATTCACGAAAAGTTTATGT	0	1
ompT	230	E. coli	pgtE	46.56	0.921	0.441	0.566	0.000	ACATATTGCTCCACTGTTTATAT	0	1
ybjX	358	E. coli	ybjX	55.56	0.784	0.000	0.433	0.000	TGATATTTCGTTGAAGTTAATGA	0	1
ybjX	360	E. coli	ybjX	55.56	0.910	0.499	0.433	0.556	CACTATTGATGTTTGGTTAAGAT	1	0
yhiE	606	E. coli	no	0.00	0.778	0.518	0.000	0.313	TGCTATTTACAAGCTGATAACAA	1	0
ompT	226	E. coli	pgtE	46.56	0.701	0.990	1.000	0.894	CACTGTTTATATTTTGTTTAGTA	0	1
yrbL	929	E. coli	yrbL	72.38	0.701	0.990	1.000	0.894	CATTGTTTAGGTTTTGTTTAAGT	1	0
ybcU	357	E. coli	no	0.00	0.701	0.990	1.000	0.894	CATTGTTTAGGGTTTGTTTAATT	1	0
pagC	1026	Salmonella	ompX	38.79	0.000	0.885	0.855	0.589	TATTATTTACGGTGTGTTTAAAC	0	1
rstA	349	Salmonella	rstA	80.75	0.000	0.754	0.702	0.589	TCTCGTTTAGAAAAGATTTATGG	1	0
ybjX	438	Salmonella	ybjX	57.14	0.661	0.930	1.000	0.853	GTTTGTTTAGATACGGTTTACTT	0	1
udg	729	Salmonella	ugd	88.92	0.661	0.930	1.000	0.853	AAATGTTTAAGCCCGGTTTAATA	1	0
mig-14	568	Salmonella	nothing	0.00	0.530	0.804	0.980	0.780	AAATGTTTAGCTTGTATTTAATG	0	1
pagC	1027	Salmonella	ompX	38.79	0.000	0.642	0.980	0.647	CTGTGTTTAGAGAGAATTTACAT	0	1
pagD	704	Salmonella	nothing	0.00	0.000	0.642	0.980	0.647	CTGTGTTTAGAGAGAATTTACAT	1	0
rstA	330	E. coli	rstA	80.75	0.000	0.550	0.805	0.569	ACCTGATGAAAACTTGTTTAGAA	1	0
mgtC	623	Salmonella	yhiD	32.21	0.000	0.630	0.906	0.574	TCATGTTTAAACACGCTTTATTT	1	0
pmrD	513	Salmonella	pmrD	59.49	0.921	0.441	0.566	0.277	CGCTATTGCCGTTTTGTTTATCC	1	0
yrbL	943	Salmonella	yrbL	72.38	0.000	1.000	0.723	0.743	TTTCGTTTAGGTTTTGTTTAAGT	1	0
phoP	277	E. coli	phoP	89.69	0.000	0.977	0.723	0.835	GCTGGTTTATTTAATGTTTACCC	1	0
phoP	604	Salmonella	phoP	89.69	0.000	0.977	0.723	0.835	TCTGGTTTATTAACTGTTTATCC	1	0
mgtA	599	Salmonella	mgtA	86.54	0.000	0.918	0.723	0.795	TCTGGTTTATCGTTGGTTTAATT	1	0
mgtA	149	E. coli	mgtA	87.61	0.000	0.918	0.723	0.795	TCTGGTTTATCGTTGGTTTAGTT	1	0
pmrD	125	E. coli	pmrD	60.00	0.000	0.699	0.578	0.590	TGCCGTTGATAAAGAGTTTATCT	1	0
yhiW	743	E. coli	adiY	40.16	0.000	0.814	0.445	0.616	CAGCGTATAGCTTATGTTTATAA	0	1
yhiW	646	E. coli	adiY	40.16	0.000	0.814	0.445	0.616	CAGCGTATAGCTTATGTTTATAA	0	1
ybjX	417	Salmonella	ybjX	57.14	0.000	0.796	0.454	0.531	TACGGTTTACTTTCTGGTTAAAA	0	1
virK	642	Salmonella	ybjX	39.08	0.701	0.755	0.433	0.419	CGCCATTGATAAACTGTTTAACA	1	0
pagP	602	Salmonella	crcA	73.68	0.000	0.804	0.723	0.922	CTCTGTTTATAGTTTGTTAAGAT	1	0
ybjX	402	Salmonella	ybjX	57.14	0.910	0.499	0.433	0.556	CTGTATTGACGATTGGTTAATGT	1	0
ugtL	883	Salmonella	nothing	0.00	0.000	0.651	0.557	0.701	CACGGTTGAGCAACTATTTACTT	0	1
slyB	352	Salmonella	slyB	70.32	0.000	0.754	0.445	0.731	CTTCGTTTAAGATTGGTTAATTA	1	0
nmpC	224	Salmonella	nmpC	81.82	0.000	0.640	0.445	0.810	CCTCGTTTAACAATGGTTGAGGA	1	0
hdeA	137	E. coli	no	0.00	0.000	0.504	0.445	0.873	TTCTGTATATGTCATGTTGATGG	0	1
hdeD	146	E. coli	no	0.00	0.000	0.504	0.445	0.873	TTCTGTATATGTCATGTTGATGG	1	0

					PhoP-Box pattern					Orientation	
										Direct	Opposite
Gene	***Code***	***Genome***	***Philogeny***	***Identity***	***M1***	***M2***	***M3***	***M4***	***Sequence***	***O1***	***O2***
hilA	822	Salmonella	ygeH	29.03	0.000	0.491	0.352	0.749	TCGGGTTTATATGTTTTTAACAT	0	1
slyB	350	E. coli	slyB	61.94	0.519	0.630	0.723	0.960	TTTTGTTTATAATTGGTTGATCC	1	0
ybjX	359	E. coli	ybjX	55.56	0.000	0.274	0.381	0.891	TTTTGTTGATATTTCGTTGAAGT	0	1
rstA	339	E. coli	rstA	80.75	0.000	0.444	0.702	0.846	ACTTGTTTAGAAACGATTGATAG	1	0
virK	643	Salmonella	ybjX	39.08	0.000	0.384	0.586	0.613	CGATGTTGTTAAACAGTTTATCA	0	1
mgtC	681	Salmonella	yhiD	32.21	0.000	0.330	0.629	0.846	TTCTGTTTAAGTTTGTTTGATAT	0	1
pipD	847	Salmonella	nothing	0.00	0.687	0.259	0.412	0.561	CTTTATTGAGGTTGTATTGATAA	0	1
pgtE	720	Salmonella	ompT	46.56	0.000	0.394	0.805	0.286	TCATGATTATAGATTGCTTATTA	1	0
mgtC	925	Salmonella	yhiD	32.21	0.000	0.000	0.761	0.355	TAATGTTTCCTTATATTTTAAAT	1	0
yeaF	469	E. coli	mipA	83.87	0.000	0.000	0.785	0.000	CATTAATTATGCAAAATTTATGG	0	1
trs5_8	354	E. coli	no	0.00	0.701	0.817	0.732	0.589	CAGTTTTTAAAAACTGTTTAAAG	0	1
yeaF	464	E. coli	mipA	83.87	0.530	0.808	0.732	0.589	ATTTGTTTAAGGAATGATTAATT	1	0
mgtC	718	Salmonella	yhiD	32.21	0.914	0.559	0.463	0.000	GTTTAGTGACGTTCTGTTTAAGT	0	1
yobG	861	Salmonella	b1826	65.96	0.575	0.650	0.331	0.000	CTACAGTTACTCCTGGTTTAAGT	1	0
yiaG	753	E. coli	yiaG	79.17	0.501	0.360	0.000	0.773	ATTTGTTGTTTCATTGTTAAAAA	1	0
ompX	242	E. coli	ompX	83.63	0.000	0.537	0.000	0.921	GCTGGTTGAGCATTTGTTGAAAA	1	0
ompX	248	Salmonella	ompX	77.78	0.000	0.262	0.000	0.833	GGCGGTTGAGGGTTCGTTGAAAA	1	0
yhdG	546	E. coli	yhdG	96.26	0.000	0.000	0.000	0.000		0	0
yaiN	356	E. coli	STM1628	52.75	0.000	0.000	0.000	0.000		0	0
pyrI	286	E. coli	pyrI	94.12	0.000	0.000	0.000	0.000		0	0
ygiW	512	E. coli	ygiW	89.23	0.000	0.000	0.000	0.000		0	0
gadB	78	E. coli	no	0.00	0.000	0.000	0.000	0.000		0	0
gadA	77	E. coli	no	0.00	0.000	0.000	0.000	0.000		0	0
dps	75	E. coli	dps	95.21	0.000	0.000	0.000	0.000		0	0
b3100	74	E. coli	yqjK	87.88	0.000	0.000	0.000	0.000		0	0
yqjE	860	E. coli	yqjE	56.92	0.000	0.000	0.000	0.000		0	0
pagK	1028	Salmonella	no	0.00	0.530	0.709	0.557	0.326	AACCATTTATAAAATATTTAACT	0	1
pdgL	1029	Salmonella	no	0.00	0.000	0.466	0.578	0.318	GAGAGTTTATATTTTGCTTATAC	0	1
pdgL	1030	Salmonella	no	0.00	0.000	0.522	0.463	0.327	TTATTTTAACCATCTGTTTAAGC	1	0
proP	1035	Salmonella	proP	78.00	0.651	0.395	0.000	0.313	ACATATTTAAACCCTGTTAGGGT	1	0
hemL	1031	E. coli	hemL	82.00	0.000	0.500	0.711	0.487	TGATGTTTGACGAGTATTTAACT	1	0
hemL	1032	E. coli	hemL	82.00	0.741	0.242	0.000	0.223	TGTTATTCATAACAAGTTAAATA	0	1
proP	1033	E. coli	proP	78.00	0.000	0.239	0.000	0.647	TTTCGTTTAGGACTCATTGATGT	0	1
proP	1034	E. coli	proP	78.00	0.000	0.274	0.557	0.356	TGAAGTTGATCACAAATTTAAAC	1	0

RNA Pol Sites

		Close II	Close I	Medium II	Medium I	Remote II	Remote I				
**Gene**	**Code**	**P1**	**P2**	**P3**	**P4**	**P5**	**P6**	**CLOSE_2_-10**	**CLOSE_2_-35**	**CLOSE_2_1**	**CLOSE_1_-10**
b2833	73	0.510	0.000	0.000	0.000	0.000	0.000	tattat	no-ttgaca	-31	no-tataat
b1825	6	0.420	0.373	0.000	0.000	0.563	0.549	no-tataat	no-ttgaca	0	no-tataat
b1826	28	0.420	0.373	0.000	0.000	0.000	0.000	TTTAAT	no-ttgaca	-37	TAAGGT
nmpC	221	0.520	0.759	0.495	0.441	0.500	0.637	TCAAAT	no-ttgaca	-30	TATAAA
slyB	351	0.310	0.000	0.616	0.667	0.469	0.549	taccgt	no-ttgaca	-43	no-tataat
ompT	230	0.310	0.771	0.000	0.373	0.000	0.000	TAAGTT	no-ttgaca	-36	TATAAA
ybjX	358	0.520	0.000	0.000	0.000	0.000	0.000	TAAAAT	no-ttgaca	-32	no-tataat
ybjX	360	0.470	0.000	0.000	0.000	0.000	0.000	TGAAAT	no-ttgaca	-32	no-tataat
yhiE	606	0.570	0.000	0.414	0.000	0.688	0.657	taggat	no-ttgaca	-37	no-tataat
ompT	226	0.420	0.410	0.000	0.627	0.000	0.000	TGTAAT	no-ttgaca	-33	TAAGTT
yrbL	929	0.410	0.000	0.000	0.000	0.000	0.000	gatcgt	no-ttgaca	-40	no-tataat
ybcU	357	0.500	0.000	0.000	0.000	0.000	0.000	TACGAT	no-ttgaca	-32	no-tataat
pagC	1026	0.670	0.000	0.000	0.000	0.000	0.000	tgttat	no-ttgaca	-41	no-tataat
rstA	349	0.680	0.819	0.414	0.000	0.000	0.000	TATGTT	TGGAAG	-43	TATGTT
ybjX	438	0.420	0.506	0.434	0.314	0.000	0.000	TGTATT	CTGACG	-48	TGTATT
udg	729	0.520	0.000	0.000	0.608	0.000	0.804	TTTTAT	no-ttgaca	-40	no-tataat
mig-14	568	0.340	0.000	0.707	0.402	0.000	0.000	ttttat	no-ttgaca	-32	no-tataat
pagC	1027	0.000	0.000	0.670	0.670	0.670	0.670	CATATT	no-ttgaca	-46	no-tataat
pagD	704	0.700	0.410	0.515	0.412	0.354	0.647	AATAAT	no-ttgaca	-31	AATATT
rstA	330	0.550	0.000	0.000	0.657	0.000	0.000	taaaaa	no-ttgaca	-38	no-tataat
mgtC	623	0.000	0.000	0.343	0.402	0.000	0.000	no-tataat	no-ttgaca	0	no-tataat
pmrD	513	0.400	0.000	0.000	0.000	0.000	0.000	TAACGT	no-ttgaca	-32	no-tataat
yrbL	943	0.630	0.000	0.000	0.529	0.000	0.000	taaatt	no-ttgaca	-36	no-tataat
phoP	277	0.700	0.000	0.000	0.000	0.000	0.000	CATAAT	no-ttgaca	-32	no-tataat
phoP	604	0.690	0.494	0.414	0.000	0.000	0.000	CATAAT	no-ttgaca	-32	TAGACT
mgtA	599	0.680	0.000	0.909	0.000	0.448	0.333	cataat	no-ttgaca	-43	no-tataat
mgtA	149	0.510	0.000	0.000	0.500	0.563	0.000	tattat	no-ttgaca	-34	no-tataat
pmrD	125	0.630	0.843	0.000	0.000	0.000	0.000	TAAATT	no-ttgaca	-32	GATAAT
yhiW	743	0.550	0.000	0.000	0.000	0.000	0.000	GAAAAT	no-ttgaca	-26	no-tataat
yhiW	646	0.340	0.410	0.515	0.402	0.781	0.667	TTTACT	GTGCGA	-48	TTTACT
ybjX	417	0.000	0.000	0.434	0.412	0.000	0.000	no-tataat	no-ttgaca	0	no-tataat
virK	642	0.000	0.000	0.606	0.304	0.000	0.000	no-tataat	no-ttgaca	0	no-tataat
pagP	602	0.510	0.000	0.475	0.000	0.000	0.000	tattat	no-ttgaca	-45	no-tataat
ybjX	402	0.430	0.000	0.000	0.000	0.000	0.000	TTCAAT	no-ttgaca	-32	no-tataat
ugtL	883	0.420	0.783	0.000	0.824	0.740	0.500	TATAAG	no-ttgaca	-37	TAAACT
slyB	352	0.510	0.000	0.000	0.765	0.000	0.304	TATGAT	no-ttgaca	-34	no-tataat
nmpC	224	0.690	0.000	0.000	0.000	0.000	0.000	tataag	no-ttgaca	-39	no-tataat
hdeA	137	0.660	0.000	0.000	0.696	0.000	0.000	TATAAC	no-ttgaca	-31	no-tataat
hdeD	146	0.680	0.000	0.545	0.333	0.427	0.480	tatttt	no-ttgaca	-43	no-tataat

RNA Pol Sites

Gene	Code	Close II<br>P1	Close I<br>P2	Medium II<br>P3	Medium I<br>P4	Remote II<br>P5	Remote I<br>P6	CLOSE_2_-10	CLOSE_2_-35	CLOSE_2_1	CLOSE_1_-10
hilA	822	0.830	0.000	0.505	0.000	0.354	0.500	tagaat	no-ttgaca	-28	no-tataat
slyB	350	0.560	0.000	0.000	0.500	0.354	0.000	tttcat	no-ttgaca	-29	no-tataat
ybjX	359	0.310	0.000	0.000	0.000	0.000	0.000	TACGTT	no-ttgaca	-35	no-tataat
rstA	339	0.670	0.807	0.000	0.000	0.000	0.000	tattgt	tagtaa	-43	tattgt
virK	643	0.000	0.000	0.606	0.304	0.000	0.000	no-tataat	no-ttgaca	0	no-tataat
mgtC	681	0.000	0.000	0.414	0.931	0.000	0.529	no-tataat	no-ttgaca	0	no-tataat
pipD	847	0.340	0.410	0.000	0.637	0.000	0.000	AAGAAT	AAGAGA	-46	AAGAAT
pgtE	720	0.530	0.000	0.374	0.422	0.000	0.333	TCAAAT	no-ttgaca	-35	no-tataat
mgtC	925	0.340	0.000	0.000	0.000	0.000	0.000	TTTACT	no-ttgaca	-24	no-tataat
yeaF	469	0.000	0.000	0.414	0.402	0.354	0.588	no-tataat	no-ttgaca	0	no-tataat
trs5_8	354	0.360	0.699	0.414	0.000	0.000	0.000	TATGGG	no-ttgaca	-45	TATTTT
yeaF	464	0.410	0.000	0.000	0.000	0.000	0.000	CAAACT	no-ttgaca	-28	no-tataat
mgtC	718	0.340	0.000	0.414	0.931	0.000	0.529	CATATT	no-ttgaca	-46	no-tataat
yobG	861	0.420	0.494	0.000	0.422	0.000	0.000	GATAAT	no-ttgaca	-32	TAAGAT
yiaG	753	0.000	0.000	0.414	0.000	0.438	0.529	no-tataat	no-ttgaca	0	no-tataat
ompX	242	0.800	0.000	0.616	0.510	0.427	0.451	taaaat	no-ttgaca	-42	no-tataat
ompX	248	0.820	0.000	0.515	0.627	0.573	0.382	taaaat	no-ttgaca	-41	no-tataat
yhdG	546	0.000	0.000	0.000	0.000	0.000	0.000	no-tataat	no-ttgaca	0	no-tataat
yaiN	356	0.000	0.000	0.000	0.000	0.000	0.000	no-tataat	no-ttgaca	0	no-tataat
pyrI	286	0.000	0.000	0.000	0.000	0.000	0.000	no-tataat	no-ttgaca	0	no-tataat
ygiW	512	0.000	0.000	0.000	0.000	0.000	0.000	no-tataat	no-ttgaca	0	no-tataat
gadB	78	0.000	0.000	0.000	0.000	0.000	0.000	no-tataat	no-ttgaca	0	no-tataat
gadA	77	0.000	0.000	0.000	0.000	0.000	0.000	no-tataat	no-ttgaca	0	no-tataat
dps	75	0.000	0.000	0.000	0.000	0.000	0.000	no-tataat	no-ttgaca	0	no-tataat
b3100	74	0.000	0.000	0.000	0.000	0.000	0.000	no-tataat	no-ttgaca	0	no-tataat
yqjE	860	0.000	0.000	0.000	0.000	0.000	0.000	no-tataat	no-ttgaca	0	no-tataat
no	1028	0.400	0.000	0.550	0.800	0.000	0.000	GATGGT	no-ttgaca	-38	no-tataat
no	1029	0.450	0.550	0.550	0.590	0.530	0.560	GATAAG	no-ttgaca	-32	GTGATA
no	1030	0.430	0.600	0.000	0.000	0.610	0.670	TAATCT	no-ttgaca	-34	CATAAT
proP	1035	0.410	0.000	0.000	0.000	0.000	0.000	CATTAA	no-ttgaca	-32	no-tataat
hemL	1031	0.790	0.500	0.400	0.400	0.000	0.000	TAACAT	no-ttgaca	-34	TAGCAG
hemL	1032	0.600	0.000	0.000	0.000	0.000	0.000	TAGCAG	no-ttgaca	-32	no-tataat
proP	1033	0.000	0.000	0.400	0.700	0.560	0.660	no-tataat	no-ttgaca	0	no-tataat
proP	1034	0.450	0.000	0.000	0.000	0.400	0.500	CATTAA	no-ttgaca	-36	no-tataat

RNA Pol Sites

Gene	***Code***	***CLOSE_1_-35***	***CLOSE_1_1***	***MEDIUM_2_-10***	***MEDIUM_2_-35***	***MEDIUM_2_1***	***MEDIUM_1_-10***	***MEDIUM_1_-35***	***MEDIUM_1_1***
b2833	73	no-ttgaca	0	no-tataat	no-ttgaca	0	no-tataat	no-ttgaca	0
b1825	6	no-ttgaca	0	no-tataat	no-ttgaca	0	no-tataat	no-ttgaca	0
b1826	28	CTAACG	-47	no-tataat	no-ttgaca	0	no-tataat	no-ttgaca	0
nmpC	221	TTGAAC	-47	TATAAA	no-ttgaca	-68	TATTTT	TTAATA	-88
slyB	351	no-ttgaca	0	tataat	no-ttgaca	-99	aataat	tagtaa	-73
ompT	230	TGTAAT	-47	no-tataat	no-ttgaca	0	AATCAT	TAGATA	-61
ybjX	358	no-ttgaca	0	no-tataat	no-ttgaca	0	no-tataat	no-ttgaca	0
ybjX	360	no-ttgaca	0	no-tataat	no-ttgaca	0	no-tataat	no-ttgaca	0
yhiE	606	no-ttgaca	0	tagtgt	no-ttgaca	-84	no-tataat	no-ttgaca	0
ompT	226	TGGAGC	-47	no-tataat	no-ttgaca	0	TATAAA	ATGTAA	-58
yrbL	929	no-ttgaca	0	no-tataat	no-ttgaca	0	no-tataat	no-ttgaca	0
ybcU	357	no-ttgaca	0	no-tataat	no-ttgaca	0	no-tataat	no-ttgaca	0
pagC	1026	no-ttgaca	0	no-tataat	no-ttgaca	0	no-tataat	no-ttgaca	0
rstA	349	TGGAAG	-43	TATTCT	no-ttgaca	-60	no-tataat	no-ttgaca	0
ybjX	438	CTGACG	-48	TTCAAT	no-ttgaca	-79	TGTGGT	TTGACG	-79
udg	729	no-ttgaca	0	no-tataat	no-ttgaca	0	TAATAT	TTCTTA	-72
mig-14	568	no-ttgaca	0	aataat	no-ttgaca	-58	tagcat	ttaagc	-65
pagC	1027	no-ttgaca	0	TATGTT	no-ttgaca	-89	TATAAT	TTTACG	-57
pagD	704	TCCAAA	-49	TATTAT	no-ttgaca	-95	TATAAA	GCGATA	-83
rstA	330	no-ttgaca	0	no-tataat	no-ttgaca	0	tattgt	tagtaa	-54
mgtC	623	no-ttgaca	0	TATAGC	CAGAAA	-82	TATTCT	GTGTGC	-95
pmrD	513	no-ttgaca	0	no-tataat	no-ttgaca	0	no-tataat	no-ttgaca	0
yrbL	943	no-ttgaca	0	no-tataat	no-ttgaca	0	taaatt	tgtcca	-50
phoP	277	no-ttgaca	0	no-tataat	no-ttgaca	0	no-tataat	no-ttgaca	0
phoP	604	TCCCCA	-46	TATTGT	no-ttgaca	-57	no-tataat	no-ttgaca	0
mgtA	599	no-ttgaca	0	tataat	no-ttgaca	-75	no-tataat	no-ttgaca	0
mgtA	149	no-ttgaca	0	no-tataat	no-ttgaca	0	tattat	tatatc	-60
pmrD	125	ATGTGA	-62	no-tataat	no-ttgaca	0	no-tataat	no-ttgaca	0
yhiW	743	no-ttgaca	0	no-tataat	no-ttgaca	0	no-tataat	no-ttgaca	0
yhiW	646	GTGCGA	-48	TATTAT	no-ttgaca	-54	TAAATT	CTGTTA	-92
ybjX	417	no-ttgaca	0	TTCAAT	no-ttgaca	-90	TGTATT	CTGACG	-59
virK	642	no-ttgaca	0	tataag	no-ttgaca	-87	tacgtt	tgtaca	-57
pagP	602	no-ttgaca	0	tcgaat	no-ttgaca	-54	no-tataat	no-ttgaca	0
ybjX	402	no-ttgaca	0	no-tataat	no-ttgaca	0	no-tataat	no-ttgaca	0
ugtL	883	TAGAAA	-45	no-tataat	no-ttgaca	0	TATTAT	CTTATA	-56
slyB	352	no-ttgaca	0	no-tataat	no-ttgaca	0	TATAAT	ATGACC	-88
nmpC	224	no-ttgaca	0	no-tataat	no-ttgaca	0	no-tataat	no-ttgaca	0
hdeA	137	no-ttgaca	0	no-tataat	no-ttgaca	0	TATAGT	TTGATT	-75
hdeD	146	no-ttgaca	0	tacaat	no-ttgaca	-54	gatact	tacaat	-79

RNA Pol Sites

Gene	*Code*	*CLOSE_1_-35*	*CLOSE_1_1*	*MEDIUM_2_-10*	*MEDIUM_2_-35*	*MEDIUM_2_1*	*MEDIUM_1_-10*	*MEDIUM_1_-35*	*MEDIUM_1_1*
hilA	822	no-ttgaca	0	cataat	no-ttgaca	-52	no-tataat	no-ttgaca	0
slyB	350	no-ttgaca	0	no-tataat	no-ttgaca	0	tatact	taacca	-86
ybjX	359	no-ttgaca	0	no-tataat	no-ttgaca	0	no-tataat	no-ttgaca	0
rstA	339	tagtaa	-43	no-tataat	no-ttgaca	0	no-tataat	no-ttgaca	0
virK	643	no-ttgaca	0	tataag	no-ttgaca	-81	tacgtt	tgtaca	-51
mgtC	681	no-ttgaca	0	TATGTT	no-ttgaca	-100	TATAAT	TTTACG	-68
pipD	847	AAGAGA	-46	no-tataat	no-ttgaca	0	CATAAT	TTTCCA	-100
pgtE	720	no-ttgaca	0	TAAAAA	no-ttgaca	-67	TAACTT	CTGAGC	-50
mgtC	925	no-ttgaca	0	no-tataat	no-ttgaca	0	no-tataat	no-ttgaca	0
yeaF	469	no-ttgaca	0	TAAATT	AAGACC	-50	TAAATT	AAGACC	-50
trs5_8	354	CTGACT	-45	TAGGAT	no-ttgaca	-87	no-tataat	no-ttgaca	0
yeaF	464	no-ttgaca	0	no-tataat	no-ttgaca	0	no-tataat	no-ttgaca	0
mgtC	718	no-ttgaca	0	TATGTT	no-ttgaca	-89	TATAAT	TTTACG	-57
yobG	861	GTAACT	-47	no-tataat	no-ttgaca	0	TAAGAT	ATGATA	-52
yiaG	753	no-ttgaca	0	tagatt	no-ttgaca	-79	no-tataat	no-ttgaca	0
ompX	242	no-ttgaca	0	aataat	no-ttgaca	-86	tggaat	tgacta	-61
ompX	248	no-ttgaca	0	aataat	no-ttgaca	-85	taaatt	tccagg	-52
yhdG	546	no-ttgaca	0	no-tataat	no-ttgaca	0	no-tataat	no-ttgaca	0
yaiN	356	no-ttgaca	0	no-tataat	no-ttgaca	0	no-tataat	no-ttgaca	0
pyrI	286	no-ttgaca	0	no-tataat	no-ttgaca	0	no-tataat	no-ttgaca	0
ygiW	512	no-ttgaca	0	no-tataat	no-ttgaca	0	no-tataat	no-ttgaca	0
gadB	78	no-ttgaca	0	no-tataat	no-ttgaca	0	no-tataat	no-ttgaca	0
gadA	77	no-ttgaca	0	no-tataat	no-ttgaca	0	no-tataat	no-ttgaca	0
dps	75	no-ttgaca	0	no-tataat	no-ttgaca	0	no-tataat	no-ttgaca	0
b3100	74	no-ttgaca	0	no-tataat	no-ttgaca	0	no-tataat	no-ttgaca	0
yqjE	860	no-ttgaca	0	no-tataat	no-ttgaca	0	no-tataat	no-ttgaca	0
no	1028	no-ttgaca	0	TATTTT	no-ttgaca	-60	TAATAT	CTGATG	-63
no	1029	AAAGAT	-49	AAAGAT	no-ttgaca	-61	TATTTT	CAGATT	-65
no	1030	CATAAT	-52	no-tataat	no-ttgaca	0	no-tataat	no-ttgaca	0
proP	1035	no-ttgaca	0	no-tataat	no-ttgaca	0	no-tataat	no-ttgaca	0
hemL	1031	TTGTTA	-44	CATGAG	no-ttgaca	-67	CATGAG	TTTTCT	-67
hemL	1032	no-ttgaca	0	no-tataat	no-ttgaca	0	no-tataat	no-ttgaca	0
proP	1033	no-ttgaca	0	CATTAA	no-ttgaca	-72	TAGGGT	TTGATC	-62
proP	1034	no-ttgaca	0	no-tataat	no-ttgaca	0	no-tataat	no-ttgaca	0

RNA Pol Sites

Gene	Code	REMOTE_2_-10	REMOTE_2_-35	REMOTE_2_1	REMOTE_1_-10	REMOTE_1_-35	REMOTE_1_1
b2833	73	no-tataat	no-ttgaca	0	no-tataat	no-ttgaca	0
b1825	6	no-tataat	no-ttgaca	0	no-tataat	no-ttgaca	0
b1826	28	no-tataat	no-ttgaca	0	no-tataat	no-ttgaca	0
nmpC	221	TAAAAA	no-ttgaca	-140	TAGGAT	TTTATT	-113
slyB	351	catatt	no-ttgaca	-133	cattat	ttataa	-119
ompT	230	no-tataat	no-ttgaca	0	no-tataat	no-ttgaca	0
ybjX	358	no-tataat	no-ttgaca	0	no-tataat	no-ttgaca	0
ybjX	360	no-tataat	no-ttgaca	0	no-tataat	no-ttgaca	0
yhiE	606	aataat	no-ttgaca	-169	taaagt	tagacg	-116
ompT	226	no-tataat	no-ttgaca	0	no-tataat	no-ttgaca	0
yrbL	929	no-tataat	no-ttgaca	0	no-tataat	no-ttgaca	0
ybcU	357	no-tataat	no-ttgaca	0	no-tataat	no-ttgaca	0
pagC	1026	no-tataat	no-ttgaca	0	no-tataat	no-ttgaca	0
rstA	349	no-tataat	no-ttgaca	0	no-tataat	no-ttgaca	0
ybjX	438	no-tataat	no-ttgaca	0	no-tataat	no-ttgaca	0
udg	729	no-tataat	no-ttgaca	0	TATACT	TTGTTT	-142
mig-14	568	no-tataat	no-ttgaca	0	no-tataat	no-ttgaca	0
pagC	1027	no-tataat	no-ttgaca	0	TACAAC	GTGACG	-207
pagD	704	AAAAAT	no-ttgaca	-128	CATAAT	TTGAAT	-145
rstA	330	no-tataat	no-ttgaca	0	no-tataat	no-ttgaca	0
mgtC	623	no-tataat	no-ttgaca	0	no-tataat	no-ttgaca	0
pmrD	513	no-tataat	no-ttgaca	0	no-tataat	no-ttgaca	0
yrbL	943	no-tataat	no-ttgaca	0	no-tataat	no-ttgaca	0
phoP	277	no-tataat	no-ttgaca	0	no-tataat	no-ttgaca	0
phoP	604	no-tataat	no-ttgaca	0	no-tataat	no-ttgaca	0
mgtA	599	cacaat	no-ttgaca	-207	gagaat	tgaacc	-149
mgtA	149	tttatt	no-ttgaca	-235	no-tataat	no-ttgaca	0
pmrD	125	no-tataat	no-ttgaca	0	no-tataat	no-ttgaca	0
yhiW	743	no-tataat	no-ttgaca	0	no-tataat	no-ttgaca	0
yhiW	646	TATACT	no-ttgaca	-161	TATAAG	TTGAAA	-188
ybjX	417	no-tataat	no-ttgaca	0	no-tataat	no-ttgaca	0
virK	642	no-tataat	no-ttgaca	0	no-tataat	no-ttgaca	0
pagP	602	no-tataat	no-ttgaca	0	no-tataat	no-ttgaca	0
ybjX	402	no-tataat	no-ttgaca	0	no-tataat	no-ttgaca	0
ugtL	883	TAAAAT	no-ttgaca	-207	TATATT	TTACTC	-161
slyB	352	no-tataat	no-ttgaca	0	TAGCGT	TTTAGC	-115
nmpC	224	no-tataat	no-ttgaca	0	no-tataat	no-ttgaca	0
hdeA	137	no-tataat	no-ttgaca	0	no-tataat	no-ttgaca	0
hdeD	146	tagatt	no-ttgaca	-138	taattt	ttgata	-163

RNA Pol Sites

Gene	Code	REMOTE_2_-10	REMOTE_2_-35	REMOTE_2_1	REMOTE_1_-10	REMOTE_1_-35	REMOTE_1_1
hilA	822	tgtatt	no-ttgaca	-123	tattat	tctatc	-132
slyB	350	tttcat	no-ttgaca	-119	no-tataat	no-ttgaca	0
ybjX	359	no-tataat	no-ttgaca	0	no-tataat	no-ttgaca	0
rstA	339	no-tataat	no-ttgaca	0	no-tataat	no-ttgaca	0
virK	643	no-tataat	no-ttgaca	0	no-tataat	no-ttgaca	0
mgtC	681	no-tataat	no-ttgaca	0	TACAAC	GTGACG	-218
pipD	847	no-tataat	no-ttgaca	0	no-tataat	no-ttgaca	0
pgtE	720	no-tataat	no-ttgaca	0	TATGAG	ATCACC	-163
mgtC	925	no-tataat	no-ttgaca	0	no-tataat	no-ttgaca	0
yeaF	469	TGTATT	no-ttgaca	-143	TAAACT	TTGGGT	-153
trs5_8	354	no-tataat	no-ttgaca	0	no-tataat	no-ttgaca	0
yeaF	464	no-tataat	no-ttgaca	0	no-tataat	no-ttgaca	0
mgtC	718	no-tataat	no-ttgaca	0	TACAAC	GTGACG	-207
yobG	861	no-tataat	no-ttgaca	0	no-tataat	no-ttgaca	0
yiaG	753	tataag	no-ttgaca	-150	tatatc	tacaaa	-117
ompX	242	taaatt	no-ttgaca	-153	aatact	tgaatg	-119
ompX	248	cattat	no-ttgaca	-118	gatgtt	ttggtg	-150
yhdG	546	no-tataat	no-ttgaca	0	no-tataat	no-ttgaca	0
yaiN	356	no-tataat	no-ttgaca	0	no-tataat	no-ttgaca	0
pyrI	286	no-tataat	no-ttgaca	0	no-tataat	no-ttgaca	0
ygiW	512	no-tataat	no-ttgaca	0	no-tataat	no-ttgaca	0
gadB	78	no-tataat	no-ttgaca	0	no-tataat	no-ttgaca	0
gadA	77	no-tataat	no-ttgaca	0	no-tataat	no-ttgaca	0
dps	75	no-tataat	no-ttgaca	0	no-tataat	no-ttgaca	0
b3100	74	no-tataat	no-ttgaca	0	no-tataat	no-ttgaca	0
yqjE	860	no-tataat	no-ttgaca	0	no-tataat	no-ttgaca	0
no	1028	no-tataat	no-ttgaca	0	no-tataat	no-ttgaca	0
no	1029	GCTAAT	no-ttgaca	-90	GCTAAT	TTAACC	-90
no	1030	CATAAT	no-ttgaca	-88	TAAAAT	CTCTCG	-88
proP	1035	no-tataat	no-ttgaca	0	no-tataat	no-ttgaca	0
hemL	1031	no-tataat	no-ttgaca	0	no-tataat	no-ttgaca	0
hemL	1032	no-tataat	no-ttgaca	0	no-tataat	no-ttgaca	0
proP	1033	TACATT	no-ttgaca	-92	TACATT	TTCAGG	-92
proP	1034	TACATT	no-ttgaca	-84	TACATT	TTCAGG	-84

		Activated/Repressed				Other TFBs						
		Activated	Repressed	AraC	ArcA	ArgR	CRP	CytR	DeoR	DnaA	FIS	FNR
Gene	Code	A1	A2	I1	I2	I3	I4	I5	I6	I7	I8	I9
b2833	73	1	0	0.5	0.5	0.5	0.5	0.5	0.5	0.5	0.5	0.5
b1825	6	1	0	0.5	0.5	0.5	0.5	0.5	0.5	0.5	0.845	0.5
b1826	28	1	0	0.5	0.5	0.5	0.465	0.5	0.5	0.5	0.5	0.5
nmpC	221	0	1	0.5	0.48	0.5	0.05	0.5	0.5	0.5	0.46	0.5
slyB	351	1	0	0.5	0.5	0.5	0.5	0.5	0.5	0.5	0.455	0.5
ompT	230	1	0	0.5	0.475	0.5	0.47	0.5	0.5	0.5	0.465	0.5
ybjX	358	1	0	0.5	0.5	0.5	0.5	0.5	0.5	0.5	0.5	0.5
ybjX	360	1	0	0.5	0.5	0.5	0.5	0.5	0.5	0.5	0.5	0.5
yhiE	606	1	0	0.5	0.5	0.5	0.455	0.5	0.5	0.5	0.465	0.46
ompT	226	1	0	0.5	0.475	0.5	0.47	0.5	0.5	0.5	0.465	0.5
yrbL	929	1	0	0.155	0.5	0.5	0.5	0.5	0.5	0.5	0.5	0.5
ybcU	357	1	0	0.185	0.5	0.5	0.5	0.5	0.5	0.5	0.5	0.455
pagC	1026	0.5	0.5	0.5	0.5	0.5	0.5	0.5	0.5	0.5	0.5	0.5
rstA	349	1	0	0.5	0.5	0.5	0.5	0.5	0.5	0.5	0.5	0.5
ybjX	438	1	0	0.5	0.5	0.5	0.47	0.5	0.5	0.5	0.09	0.5
udg	729	1	0	0.5	0.47	0.5	0.5	0.5	0.5	0.5	0.5	0.5
mig-14	568	1	0	0.5	0.475	0.5	0.215	0.5	0.5	0.5	0.5	0.5
pagC	1027	1	0	0.5	0.5	0.5	0.5	0.5	0.5	0.5	0.5	0.5
pagD	704	1	0	0.5	0.475	0.5	0.535	0.5	0.5	0.5	0.46	0.5
rstA	330	1	0	0.5	0.5	0.5	0.5	0.5	0.5	0.5	0.5	0.5
mgtC	623	1	0	0.5	0.5	0.5	0.5	0.5	0.5	0.5	0.195	0.5
pmrD	513	1	0	0.5	0.5	0.5	0.47	0.5	0.5	0.5	0.17	0.5
yrbL	943	1	0	0.5	0.5	0.5	0.46	0.5	0.5	0.12	0.5	0.205
phoP	277	1	0	0.5	0.5	0.5	0.5	0.5	0.5	0.5	0.5	0.5
phoP	604	1	0	0.5	0.5	0.5	0.5	0.5	0.5	0.5	0.5	0.5
mgtA	599	1	0	0.5	0.5	0.5	0.47	0.5	0.5	0.5	0.5	0.5
mgtA	149	1	0	0.5	0.5	0.5	0.465	0.5	0.5	0.5	0.5	0.5
pmrD	125	1	0	0.5	0.5	0.5	0.5	0.5	0.5	0.5	0.5	0.5
yhiW	743	0	0	0.5	0.5	0.5	0.53	0.5	0.5	0.07	0.455	0.5
yhiW	646	0.5	0.5	0.5	0.5	0.5	0.53	0.5	0.5	0.07	0.455	0.5
ybjX	417	1	0	0.5	0.5	0.5	0.47	0.5	0.5	0.5	0.09	0.5
virK	642	1	0	0.5	0.5	0.5	0.46	0.5	0.5	0.5	0.455	0.5
pagP	602	0.5	0.5	0.5	0.5	0.5	0.455	0.5	0.5	0.5	0.135	0.5
ybjX	402	1	0	0.5	0.5	0.5	0.47	0.5	0.5	0.5	0.09	0.5
ugtL	883	0.5	0.5	0.5	0.47	0.5	0.47	0.465	0.5	0.5	0.46	0.5
slyB	352	0.5	0.5	0.5	0.5	0.5	0.5	0.5	0.5	0.5	0.455	0.5
nmpC	224	0	1	0.5	0.53	0.5	0.54	0.5	0.5	0.5	0.535	0.54
hdeA	137	1	0	0.5	0.5	0.5	0.46	0.5	0.5	0.5	0.5	0.5
hdeD	146	0.5	0.5	0.5	0.5	0.5	0.46	0.5	0.5	0.5	0.5	0.5

		Activated/Repressed		Other TFBs								
		Activated	Repressed	AraC	ArcA	ArgR	CRP	CytR	DeoR	DnaA	FIS	FNR
Gene	Code	A1	A2	I1	I2	I3	I4	I5	I6	I7	I8	I9
hilA	822	0	1	0.155	0.5	0.5	0.465	0.5	0.5	0.5	0.54	0.5
slyB	350	1	0	0.5	0.5	0.5	0.5	0.5	0.5	0.5	0.455	0.5
ybjX	359	1	0	0.5	0.5	0.5	0.5	0.5	0.5	0.5	0.5	0.5
rstA	339	0.5	0.5	0.5	0.5	0.5	0.5	0.5	0.5	0.5	0.5	0.5
virK	643	1	0	0.5	0.5	0.5	0.46	0.5	0.5	0.5	0.455	0.5
mgtC	681	1	0	0.5	0.5	0.5	0.5	0.5	0.5	0.5	0.195	0.5
pipD	847	0.5	0.5	0.5	0.5	0.5	0.5	0.5	0.5	0.5	0.5	0.5
pgtE	720	1	0	0.5	0.5	0.5	0.455	0.5	0.5	0.5	0.185	0.5
mgtC	925	0	1	0.5	0.5	0.5	0.5	0.5	0.5	0.5	0.195	0.5
yeaF	469	0	1	0.5	0.5	0.5	0.54	0.5	0.5	0.5	0.465	0.5
trs5_8	354	0.5	0.5	0.5	0.5	0.5	0.46	0.5	0.5	0.5	0.5	0.5
yeaF	464	0	1	0.5	0.5	0.5	0.54	0.5	0.5	0.5	0.465	0.5
mgtC	718	1	0	0.5	0.5	0.5	0.5	0.5	0.5	0.5	0.195	0.5
yobG	861	1	0	0.5	0.5	0.5	0.5	0.5	0.5	0.5	0.46	0.5
yiaG	753	0.5	0.5	0.5	0.48	0.5	0.53	0.5	0.5	0.5	0.5	0.5
ompX	242	1	0	0.5	0.5	0.5	0.455	0.5	0.5	0.5	0.455	0.545
ompX	248	0.5	0.5	0.5	0.5	0.165	0.465	0.5	0.5	0.5	0.46	0.545
yhdG	546	0	0	0.5	0.52	0.5	0.545	0.54	0.825	0.5	0.95	0.5
yaiN	356	0	0	0.5	0.5	0.5	0.5	0.5	0.5	0.5	0.5	0.5
pyrI	286	0	0	0.5	0.5	0.5	0.5	0.5	0.5	0.5	0.5	0.5
ygiW	512	0	0	0.5	0.5	0.5	0.5	0.5	0.5	0.5	0.5	0.5
gadB	78	0	0	0.5	0.53	0.5	0.53	0.5	0.5	0.885	0.54	0.5
gadA	77	0	0	0.5	0.54	0.5	0.54	0.5	0.5	0.5	0.5	0.5
dps	75	0	0	0.5	0.5	0.5	0.5	0.5	0.5	0.5	0.81	0.5
b3100	74	0	0	0.5	0.5	0.5	0.5	0.5	0.5	0.5	0.5	0.5
yqjE	860	0	0	0.5	0.5	0.5	0.5	0.5	0.5	0.5	0.5	0.5
no	1028	1	0	0.5	0.770	0.5	0.5	0.5	0.5	0.5	0.740	0.5
no	1029	0.5	0.5	0.5	0.5	0.5	0.700	0.5	0.5	0.5	0.5	0.5
no	1030	1	0	0.5	0.5	0.5	0.700	0.5	0.5	0.5	0.5	0.5
proP	1035	1	0	0.5	0.5	0.5	0.5	0.5	0.5	0.5	0.780	0.5
hemL	1031	1	0	0.5	0.5	0.5	0.5	0.5	0.5	0.5	0.5	0.5
hemL	1032	0.5	0.5	0.5	0.5	0.5	0.5	0.5	0.5	0.5	0.5	0.5
proP	1033	1	0	0.5	0.5	0.5	0.760	0.5	0.5	0.5	0.800	0.5
proP	1034	0.5	0.5	0.5	0.5	0.5	0.760	0.5	0.5	0.5	0.800	0.5

Other TFBs

		FruR	Fur	GlpR	IHF	Lrp	MalT	MelR	NarL	OmpR	OxyR	RcsU	RhaS
**Gene**	**Code**	**I10**	**I11**	**I12**	**I13**	**I14**	**I15**	**I16**	**I17**	**I18**	**I19**	**I20**	**I21**
b2833	73	0.5	0.5	0.5	0.5	0.455	0.5	0.5	0.5	0.5	0.5	0.5	0.5
b1825	6	0.5	0.5	0.5	0.5	0.5	0.5	0.5	0.5	0.5	0.5	0.5	0.5
b1826	28	0.5	0.5	0.5	0.5	0.53	0.5	0.5	0.5	0.5	0.5	0.5	0.5
nmpC	221	0.5	0.5	0.5	0.05	0.205	0.5	0.5	0.5	0.95	0.5	0.5	0.5
slyB	351	0.5	0.5	0.5	0.455	0.215	0.5	0.5	0.5	0.5	0.5	0.5	0.5
ompT	230	0.5	0.5	0.5	0.2	0.46	0.5	0.5	0.2	0.12	0.5	0.5	0.5
ybjX	358	0.5	0.5	0.5	0.455	0.47	0.5	0.5	0.215	0.5	0.5	0.5	0.5
ybjX	360	0.5	0.5	0.5	0.455	0.47	0.5	0.5	0.215	0.5	0.5	0.5	0.5
yhiE	606	0.5	0.5	0.5	0.825	0.19	0.5	0.5	0.46	0.5	0.5	0.5	0.5
ompT	226	0.5	0.5	0.5	0.2	0.46	0.5	0.5	0.2	0.12	0.5	0.5	0.5
yrbL	929	0.5	0.5	0.5	0.5	0.47	0.5	0.5	0.2	0.5	0.5	0.5	0.455
ybcU	357	0.5	0.5	0.5	0.175	0.455	0.5	0.5	0.46	0.5	0.5	0.5	0.5
pagC	1026	0.5	0.5	0.5	0.86	0.81	0.5	0.5	0.5	0.14	0.5	0.5	0.5
rstA	349	0.5	0.5	0.5	0.5	0.46	0.5	0.5	0.5	0.5	0.5	0.5	0.5
ybjX	438	0.5	0.5	0.5	0.5	0.455	0.5	0.5	0.5	0.5	0.5	0.5	0.5
udg	729	0.5	0.5	0.5	0.545	0.545	0.5	0.5	0.21	0.5	0.5	0.15	0.5
mig-14	568	0.5	0.5	0.5	0.455	0.455	0.5	0.5	0.5	0.08	0.5	0.5	0.5
pagC	1027	0.5	0.5	0.5	0.86	0.81	0.5	0.5	0.5	0.14	0.5	0.5	0.5
pagD	704	0.5	0.5	0.5	0.205	0.46	0.5	0.5	0.185	0.5	0.5	0.5	0.5
rstA	330	0.5	0.5	0.5	0.5	0.5	0.5	0.5	0.5	0.5	0.5	0.5	0.5
mgtC	623	0.5	0.5	0.5	0.455	0.46	0.5	0.5	0.5	0.5	0.5	0.5	0.5
pmrD	513	0.5	0.5	0.5	0.5	0.46	0.5	0.5	0.2	0.5	0.5	0.5	0.5
yrbL	943	0.5	0.5	0.5	0.45	0.5	0.5	0.5	0.5	0.5	0.5	0.5	0.5
phoP	277	0.5	0.5	0.5	0.205	0.46	0.5	0.5	0.455	0.5	0.5	0.5	0.5
phoP	604	0.5	0.5	0.5	0.455	0.455	0.5	0.5	0.5	0.5	0.5	0.5	0.5
mgtA	599	0.5	0.5	0.5	0.46	0.545	0.5	0.815	0.5	0.5	0.5	0.5	0.5
mgtA	149	0.5	0.5	0.5	0.455	0.455	0.82	0.5	0.5	0.5	0.5	0.5	0.5
pmrD	125	0.5	0.5	0.5	0.46	0.46	0.5	0.5	0.455	0.5	0.5	0.5	0.5
yhiW	743	0.5	0.5	0.5	0.17	0.545	0.5	0.5	0.46	0.5	0.5	0.5	0.5
yhiW	646	0.5	0.5	0.5	0.17	0.545	0.5	0.5	0.46	0.5	0.5	0.5	0.5
ybjX	417	0.5	0.5	0.5	0.5	0.455	0.5	0.5	0.5	0.5	0.5	0.5	0.5
virK	642	0.5	0.5	0.5	0.455	0.535	0.5	0.5	0.87	0.13	0.5	0.5	0.5
pagP	602	0.5	0.5	0.5	0.145	0.145	0.5	0.5	0.175	0.5	0.5	0.5	0.5
ybjX	402	0.5	0.5	0.5	0.5	0.455	0.5	0.5	0.5	0.5	0.5	0.5	0.5
ugtL	883	0.5	0.5	0.45	0.455	0.46	0.5	0.5	0.455	0.5	0.5	0.5	0.5
slyB	352	0.5	0.5	0.5	0.46	0.46	0.5	0.5	0.175	0.135	0.5	0.5	0.5
nmpC	224	0.5	0.175	0.5	0.175	0.545	0.5	0.5	0.545	0.5	0.5	0.5	0.5
hdeA	137	0.5	0.5	0.5	0.175	0.21	0.5	0.5	0.155	0.5	0.5	0.5	0.5
hdeD	146	0.5	0.5	0.5	0.16	0.195	0.5	0.5	0.5	0.5	0.5	0.5	0.5

Other TFBs

Gene	Code	FruR I10	Fur I11	GlpR I12	IHF I13	Lrp I14	MalT I15	MelR I16	NarL I17	OmpR I18	OxyR I19	RcsU I20	RhaS I21
hilA	822	0.5	0.5	0.5	0.18	0.54	0.5	0.5	0.19	0.5	0.5	0.5	0.5
slyB	350	0.5	0.5	0.5	0.455	0.215	0.5	0.5	0.5	0.5	0.5	0.5	0.5
ybjX	359	0.5	0.5	0.5	0.455	0.47	0.5	0.5	0.215	0.5	0.5	0.5	0.5
rstA	339	0.5	0.5	0.5	0.5	0.5	0.5	0.5	0.5	0.5	0.5	0.5	0.5
virK	643	0.5	0.5	0.5	0.455	0.535	0.5	0.5	0.87	0.13	0.5	0.5	0.5
mgtC	681	0.5	0.5	0.5	0.455	0.46	0.5	0.5	0.5	0.5	0.5	0.5	0.5
pipD	847	0.5	0.5	0.5	0.46	0.465	0.5	0.5	0.455	0.5	0.5	0.5	0.5
pgtE	720	0.5	0.5	0.5	0.455	0.46	0.5	0.5	0.195	0.5	0.5	0.5	0.5
mgtC	925	0.5	0.5	0.5	0.455	0.46	0.5	0.5	0.5	0.5	0.5	0.5	0.5
yeaF	469	0.5	0.5	0.5	0.16	0.455	0.5	0.5	0.455	0.5	0.5	0.5	0.145
trs5_8	354	0.5	0.5	0.5	0.21	0.535	0.5	0.5	0.5	0.5	0.5	0.5	0.5
yeaF	464	0.5	0.5	0.5	0.16	0.455	0.5	0.5	0.455	0.5	0.5	0.5	0.145
mgtC	718	0.5	0.5	0.5	0.455	0.46	0.5	0.5	0.5	0.5	0.5	0.5	0.5
yobG	861	0.5	0.5	0.5	0.46	0.465	0.5	0.5	0.5	0.5	0.5	0.5	0.5
yiaG	753	0.875	0.5	0.5	0.46	0.46	0.5	0.5	0.5	0.5	0.5	0.5	0.5
ompX	242	0.5	0.5	0.5	0.805	0.785	0.5	0.5	0.46	0.87	0.5	0.5	0.5
ompX	248	0.5	0.5	0.5	0.805	0.785	0.5	0.5	0.455	0.85	0.5	0.5	0.5
yhdG	546	0.5	0.5	0.5	0.79	0.8	0.5	0.5	0.545	0.5	0.5	0.5	0.5
yaiN	356	0.5	0.5	0.5	0.5	0.54	0.5	0.5	0.545	0.5	0.5	0.5	0.5
pyrI	286	0.5	0.5	0.5	0.5	0.5	0.5	0.5	0.5	0.5	0.5	0.5	0.5
ygiW	512	0.5	0.5	0.545	0.545	0.545	0.5	0.5	0.5	0.85	0.5	0.5	0.5
gadB	78	0.5	0.5	0.5	0.8	0.815	0.5	0.5	0.5	0.9	0.5	0.5	0.5
gadA	77	0.5	0.5	0.5	0.875	0.825	0.5	0.5	0.5	0.5	0.5	0.5	0.5
dps	75	0.5	0.5	0.5	0.95	0.535	0.5	0.5	0.545	0.5	0.95	0.5	0.5
b3100	74	0.5	0.5	0.5	0.5	0.5	0.5	0.5	0.5	0.5	0.5	0.5	0.5
yqjE	860	0.5	0.5	0.5	0.5	0.5	0.5	0.5	0.5	0.5	0.5	0.5	0.5
no	1028	0.5	0.890	0.5	0.5	0.790	0.5	0.5	0.710	0.5	0.5	0.5	0.5
no	1029	0.5	0.5	0.5	0.5	0.5	0.5	0.5	0.5	0.5	0.5	0.5	0.5
no	1030	0.5	0.5	0.5	0.5	0.5	0.5	0.5	0.5	0.5	0.5	0.5	0.5
proP	1035	0.5	0.5	0.5	0.5	0.670	0.5	0.5	0.5	0.5	0.5	0.5	0.5
hemL	1031	0.5	0.5	0.5	0.5	0.500	0.5	0.5	0.5	0.5	0.5	0.5	0.5
hemL	1032	0.5	0.5	0.5	0.5	0.500	0.5	0.5	0.5	0.5	0.5	0.5	0.5
proP	1033	0.5	0.5	0.5	0.5	0.670	0.5	0.5	0.5	0.5	0.5	0.5	0.5
proP	1034	0.5	0.5	0.5	0.5	0.670	0.5	0.5	0.5	0.5	0.5	0.5	0.5

		Other TFBs		
		YgiX	***PmrA***	***SlyA***
Gene	***Code***	***I22***	***I23***	***I24***
b2833	73	0.5	0.5	0.5
b1825	6	0.5	0.5	0.5
b1826	28	0.5	0.5	0.44
nmpC	221	0.5	0.5	0.21
slyB	351	0.5	0.5	0.5
ompT	230	0.5	0.5	0.4
ybjX	358	0.5	0.5	0.5
ybjX	360	0.5	0.5	0.5
yhiE	606	0.5	0.165	0.133
ompT	226	0.5	0.5	0.4
yrbL	929	0	0.5	0.5
ybcU	357	0.5	0.5	0.5
pagC	1026	0.5	0.5	0.88
rstA	349	0.5	0.5	0.5
ybjX	438	0.5	0.5	0.5
udg	729	0	0.5	0.5
mig-14	568	0.5	0.5	0.78
pagC	1027	0.5	0.5	0.88
pagD	704	0.5	0.5	0.88
rstA	330	0.5	0.5	0.5
mgtC	623	0.5	0.5	0.7
pmrD	513	0	0.5	0.5
yrbL	943	0	0.5	0.5
phoP	277	0.5	0.5	0.5
phoP	604	0.5	0.5	0.5
mgtA	599	0.5	0.5	0.5
mgtA	149	0.5	0.5	0.5
pmrD	125	0.5	0.5	0.5
yhiW	743	0.5	0.5	0.5
yhiW	646	0.5	0.5	0.5
ybjX	417	0.5	0.5	0.5
virK	642	0.5	0.5	0.7
pagP	602	0.5	0.5	0.5
ybjX	402	0.5	0.5	0.5
ugtL	883	0.5	0.5	0.86
slyB	352	0.5	0.5	0.5
nmpC	224	0.5	0.5	0.24
hdeA	137	0.5	0.17	0.5
hdeD	146	0.5	0.1	0.5

Other TFBs

**Gene**	**Code**	***YgiX* I22**	***PmrA* I23**	***SlyA* I24**
hilA	822	0.5	0.5	0.3
slyB	350	0.5	0.5	0.5
ybjX	359	0.5	0.5	0.5
rstA	339	0.5	0.5	0.5
virK	643	0.5	0.5	0.59
mgtC	681	0.5	0.5	0.3
pipD	847	0.5	0.5	0.4
pgtE	720	0.5	0.5	0.69
mgtC	925	0.5	0.5	0.7
yeaF	469	0	0.5	0.5
trs5_8	354	0	0.5	0.4
yeaF	464	0	0.5	0.5
mgtC	718	0.5	0.5	0.3
yobG	861	0.5	0.5	0.5
yiaG	753	0.5	0.5	0.5
ompX	242	0.5	0.5	0.5
ompX	248	0.5	0.5	0.5
yhdG	546	0.5	0.5	0.4
yaiN	356	0.5	0.5	0.5
pyrI	286	0.5	0.5	0.5
ygiW	512	1	0.5	0.5
gadB	78	0.5	0.99	0.5
gadA	77	0.5	0.99	0.4
dps	75	0.5	0.5	0.5
b3100	74	0.5	0.5	0.5
yqjE	860	0.5	0.5	0.5
no	1028	0.5	0.78	0.710
no	1029	0.5	0.5	0.5
no	1030	0.5	0.5	0.5
proP	1035	0.5	0.5	0.5
hemL	1031	0.5	0.5	0.4
hemL	1032	0.5	0.5	0.4
proP	1033	0.5	0.5	0.5
proP	1034	0.5	0.5	0.5

**Table S2:** Bacterial strains and plasmids used in this study		
Strain or plasmid	***Description***	***Ref. or source***
S. enterica serovar Typhimurium		
14028s	wild-type	(20)
E. coli		
DH5α	F^- *sup*E44 Δ*lac*U169 (ϕ80 *lacZ*ΔM15) *hsd*R17 *rec*A1 *end*A1 *gyr*A96 *thi*-1 *rel*A1	(21)
Plasmids		
pMS201	pSC101*ori* Km^R, promoterless GFP*mut2* vector	(22)
pSGF101	pSC101*ori* Km^R, P*phoP*-GFP*mut2*	This work
pSGF102	pSC101*ori* Km^R, P*pmrD*-GFP*mut2*	This work
pSGF103	pSC101*ori* Km^R, P*slyB*-GFP*mut2*	This work
pSGF106	pSC101*ori* Km^R, P*ugtL*-GFP*mut2*	This work
pSGF107	pSC101*ori* Km^R, P*pagC*-GFP*mut2*	This work
pSGF109	pSC101*ori* Km^R, P*pagK*-GFP*mut2*	This work
pSGF111	pSC101*ori* Km^R, P*yobG*-GFP*mut2*	This work
pSGF112	pSC101*ori* Km^R, P*ugd*-GFP*mut2*	This work

**Table S3:** Primers used to construct the promoters in GFP reporter plasmids		
Primers	***Promoters***	***Sequences***
4811	*phoP*	5′ CCGCTCGAGAAGAGTTGACCCGTGGCAAGCGTG 3′
4700	*phoP*	5′ CGGGATCCGTGGCGTAATAATGCATTATCCTC 3′
4740	*pmrD*	5′ TTAGGGATCCGCCGCTATCGCACAGCAC 3′
4741	*pmrD*	5′ AAGATCTCGAGTTTGGTCATCGTCCCGTTG 3′
5611	*yobG*	5′ CGGGATCCAACAGGCATACTACCACCACGATG 3′
5612	*yobG*	5′ CGGCTCGAGATGCCATAGCCGCTCATCAGCAG 3′
4742	*slyB*	5′ TCGGATCCGGTATAAACATCACCCGAAAG 3′
4743	*slyB*	5′ CGGCTCGAGTTCATACCTCTTCCTTTTAAGC 3′
4805	*pagC*	5′ CGGGATCCGTTAGTATCGGCCTGTGCAAC 3′
4806	*pagC*	5′ CCGCTCGAGGTTTTACTACCAAAATCGTAG 3′
5227	*pagK*	5′ CGGGATCCTGAAGATGGTAATATTAAAACC 3′
5228	*pagK*	5′ CGGCTCGAGAACAGCAGAAGAGTTGTTATG 3′
4885	*ugtL*	5′ CGGGATCCTGACTGCAAAATGCCCCAGGATGC 3′
4886	*ugtL*	5′ CCGCTCGAGTCAAATCGACCCGTAGCTTTAGTC 3′
4883	*ugd*	5′ CGGGATCCGCAATAAGCAAACCATTAGACAAGC 3′
4884	*ugd*	5′ CCGCTCGAGTGCCGACATTCTCTGCGGCAGTG 3′
